# AI-guided optimization of traditional bulgur pilafs: enhancing sensory and bioactive properties through RSM-PSO modeling

**DOI:** 10.3389/fnut.2025.1658452

**Published:** 2025-10-24

**Authors:** Sinem Türk Aslan, Melikenur Türkol, Seydi Yıkmiş, Mehmet Ali Şimşek, Moneera O. Aljobair, Emad Karrar, Nazlı Tokatlı, Isam A. Mohamed Ahmed

**Affiliations:** ^1^Culinary Program, Department of Hotel, Restaurant and Catering Services, Tavas Vocational School, Pamukkale University, Denizli, Türkiye; ^2^Department of Nutrition and Dietetics, Tekirdaˇ g Namik Kemal University, Tekirdaˇ g, Türkiye; ^3^Department of Food Technology, Tekirdag Namık Kemal University, Tekirdag, Türkiye; ^4^Department of Computer Technologies, Vocational School of Technical Sciences, Tekirdag Namik Kemal University, Tekirdag, Türkiye; ^5^Department of Sports Health, College of Sports Sciences and Physical Activity, Princess Nourah Bint Abdulrahman University, Riyadh, Saudi Arabia; ^6^Department of Plant Sciences, North Dakota State University, Fargo, ND, United States; ^7^Department of Computer Engineering, Faculty of Engineering and Natural Sciences, Istanbul Health and Technology University, Istanbul, Türkiye; ^8^Department of Food Science and Nutrition, College of Food and Agricultural Sciences, King Saud University, Riyadh, Saudi Arabia

**Keywords:** particle swarm optimization (PSO), bulgur pilaf, sensory analysis, geographical indication, personalized nutrition

## Abstract

This study aimed to enhance the sensory and bioactive properties of pilafs prepared from three geographically indicated bulgur varieties–Siyez, Firik, and Karakilçik–through an AI-guided optimization approach combining Response Surface Method (RSM) and Particle Swarm Optimization (PSO). Different bulgur (130–150 g) and water (350–450 mL) ratios were tested to determine optimal formulations. Sensory evaluation revealed that Firik bulgur pilaf achieved the highest overall acceptability (8.49), while Karakilçik bulgur pilaf scored highest in color (7.68) and aroma (8.58), and Siyez bulgur pilaf received the highest taste score (7.50). In terms of bioactive properties, Karakilçik bulgur pilaf showed the highest antioxidant capacity (75.57% DPPH radical scavenging activity), whereas Firik bulgur pilaf had the highest total phenolic (842.39 mg GAE/kg) and flavonoid contents (6.38 mg CE/g). Color analysis indicated that Siyez bulgur pilaf had the lightest color (*L* = 52.18), while Firik pilaf exhibited the most intense red hue (*a* = 8.12) and Karakilçik pilaf the darkest appearance (*L* = 35.42). PSO-based validation confirmed the accuracy of RSM models by reaching global optima within 40 iterations and minimal deviation from experimental values. This is the first study to apply an integrated RSM–PSO modeling approach to traditional bulgur pilafs, enabling the prediction and optimization of their sensory and bioactive characteristics. The results provide a novel framework for enhancing the nutritional value and consumer appeal of heritage cereal-based foods and support the development of standardized, functional bulgur products for the food industry.

## 1 Introduction

Bulgur is a semi-prepared food traditionally obtained by washing, boiling, sun drying, sorting, and crushing wheat into small sizes in the mill (1, 2). The quality of bulgur is related to the type of wheat. In particular, bulgur obtained from hard durum wheat (*Triticum durum*) is richer in protein and is of better quality. Bread wheat (*T. aestivum*), durum wheat (*T. durum*), Siyez wheat (*T. monococcum*), and emmer wheat (*T. dicoccum*) are generally used in bulgur production. Durum wheat is generally preferred in bulgur production due to its hardness and amber color (3, 4). There are durum wheat varieties such as Ankara-98, Red Kazmalı wheat, Levante, Red wheat, Burgaz, Kızıltan, Kunduru-1149, Variety 1252, Gediz 75, Mirzabey, Diyarbakır-81, Dicle-74, Fırat- 93, and Kunduru grown in Anatolian soils (1, 5). Bulgur is also rich in dietary fiber and vitamin B content (6). In addition to its nutritional properties, bulgur has anti-carcinogenic, anti-microbial, anti-diabetic, and anti-oxidative properties. Bulgur reduces the risk of chronic diseases, supports weight loss, and improves digestion and intestinal health (7).

The crushed bulgur is passed through sieves with different hole sizes (0.5–3.5 mm) and classified as pilaf and meatball bulgur according to their sizes (4). Bulgur obtained in different sizes has a neutral taste. It is used to prepare many dishes, such as salads, soups, pilaf, and meatballs, as it easily adapts to flavors such as sweet, sour, bitter, and salty (8, 9).

Wheat is a remarkable plant that grows for 1 year and is one of nearly 12,000 plant taxa in our country. In Türkiye, more than 20 wild wheat and more than 400 improved wheat varieties are cultivated (10). Siyez and Karakilçik wheat, which are ancestral wheat varieties, have been carried to the present day as the most primitive form of wheat. Primitive wheat is resistant to natural conditions and diseases such as frost, drought, and temperature during growth due to its high salt content, and can grow even in infertile soils. For this reason, it is preferred for wheat cultivation. They are rich in fiber due to their spelled structure. In addition to these features, the use of Siyez and Karakilçik wheat is gradually increasing today because their unique flavors are desirable, their nutritional contents are more prosperous than other wheat varieties, and their glycaemic indices are low (11–15). Siyez wheat is reported to be beneficial against blood cholesterol, has low allergenicity and high protein content, and is rich in lutein, a yellow-pigmented carotenoid (11, 12). The most important characteristic of Karakilçik wheat is that it is darker and more complex than other wheat. Karakilçik wheat contains less gluten than other wheat. The flavor of Karakilçik wheat, which is durum wheat, is distinct and more nutritious (13).

Firik, a traditional wheat type known as freekeh, is obtained by roasting the wheat that has not completed its maturity and is in the milky stage with the help of fire. The harvest time of durum wheat to be used in fabric production is also one of the factors determining the quality. When the harvest is done early, the grain spike burns, and the grains remain small. When the harvest is late, the desired aroma and color formation are not at the desired level. Therefore, it should be harvested when the spike and leaf parts are green. Firik, which has green-brown tones, contains a sooty aroma. While the quality of the firik is determined by appropriate burning and drying processes, the wheat variety preferred for making firik also determines this quality. In terms of nutritional content, it has been determined that firik has a richer nutritional content compared to other wheat because it contains approximately 77% total carbohydrate, 13% protein, 2% fat, high amounts of dietary fiber, magnesium, potassium, and calcium and small amounts of vitamins A, B1, B2, C and E (16).

These geographically indicated bulgurs are obtained from a specific geographical area and carry the traditional characteristics of that region. Thus, the characteristics of these bulgur types are accurately transferred to future generations. In addition, these bulgur types with geographical indication are sold at a higher price than other bulgur types and provide quality assurance. In this way, the development of the local people and the preservation of regional skills and traditions are ensured (9).

In a previous study (16), the color and quality characteristics of bulgur samples made from Siyez and durum wheat were evaluated. These included yield, optimum cooking time, volume expansion, and water absorption, using six different preparation methods that combined three cooking techniques (traditional, microwave, and autoclave) with two drying methods (microwave and hot air). Sensory evaluation and texture profile analysis were also conducted on the bulgur samples (17). Pekkirişçi et al. (18) obtained samples of 10 Siyez and 10 Firik bulgurs from markets in different Turkish regions. The samples’ physical and nutritional properties were then evaluated (18). To evaluate differences in the flavor and aroma characteristics of cooked wheat grains, Starr et al. (19) conducted a sensory evaluation using 24 wheat samples representing various species, landraces, and cultivars, and found that many wheat varieties exhibited significant differences in their sensory profiles (19).

The demand for bulgur in the world is constantly increasing, in parallel with the increase in the world population, the interest of the globalizing world in different food habits, and the rise of the “healthy nutrition” sector, with the features of bulgur such as its cheapness, nutritiousness, easy preparation, ability to be used in different types of dishes and can be used for a long time (20). With the development of the ready-made food industry, consumers’ demands may shift towards healthy, traditional and delicious products. Pilaf produced from geographically indicated bulgur is also suitable for meeting this need in the ready-made food industry.

Optimization involves maximizing the benefit of a product, process, or system. The response surface method is the sum of statistical and mathematical techniques used in process development, improvement, and optimization. It determines the effects of multiple factors and their interactions on one or more response-response variables (21–26).

Response surface method uses maths and stats to estimate the influence of independent parameters and determine their optimal values (27). Particle swarm optimization (PSO) was also included in the study to validate the prediction surfaces obtained with RSM and to reliably determine the global optimum. The stochastic nature of PSO provides the ability to converge in multivariate food systems in a short time without getting stuck in local maxima and allows the peaks predicted by RSM to be tested independently. Thus, the RSM-PSO composite approach was able to reliably determine the optimal processing parameters for the criteria taste, color, smell and general acceptance for three different bulgur types.

**FIGURE 1:**
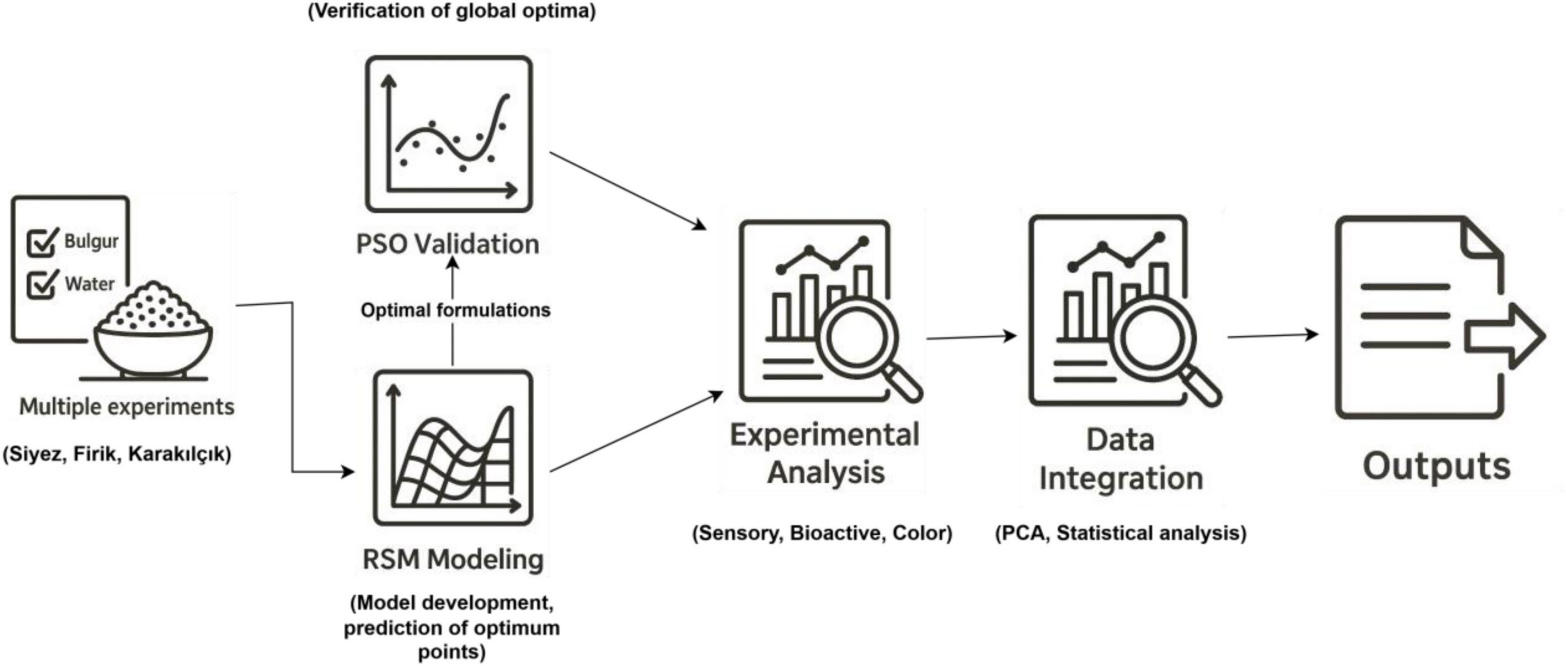
Workflow diagram of the study.

In Türkiye, geographical indications (GIs) are protected under the Industrial Property Law No. 6769, which aims to safeguard the unique characteristics of traditional products originating from specific regions. The Turkish Patent and Trademark Office (TURKPATENT) manages the registration and regulation of GIs. This study’s selected bulgur varieties–Siyez, Firik, and Karakilçik–represent traditional and regionally significant products. Siyez bulgur is recognized as a GI product associated with the Kastamonu region, while Firik bulgur is a registered GI product linked to the Hatay region. Karakilçik bulgur, although not formally registered, is widely acknowledged as a traditional variety specific to the Amik Plain (Hatay region). Including these varieties aligns with Türkiye’s GI regulations, supporting the preservation of regional heritage, cultural identity, and sustainable agricultural practices (28). In this study, three geographically indicated bulgur varieties (Siyez, Firik, and Karakilçik) were used, and the amounts of bulgur and water were determined by the RSM method. The effects of the varieties on the quality of bulgur pilafs were tried to be revealed with the formulations. This study investigated the impact of different amounts of water and wheat varieties on bulgur pilafs’ color, sensory, and bioactive properties. Since wheat varieties have not been examined in the context of pilaf quality/RSM studies, an original and innovative approach has been presented with this research. This study is the first to apply AI-based optimization methods (RSM–PSO) to traditional bulgur pilafs prepared from geographically indicated Siyez, Firik, and Karakilçik varieties. In the existing literature, the use of AI-driven multi-model optimization approaches for improving the quality parameters of traditional cereal-based products is extremely limited, and this study demonstrates their applicability to bulgur pilafs. This innovative approach not only enhances the functional and sensory qualities of bulgur pilafs but also provides a novel methodological framework for functional food development and personalized nutrition. The workflow steps of the study were carried out in accordance with the flowchart shown in Figure 1.

## 2 Materials and methods

### 2.1 Materials

In this study, Siyez (Anatolian flavors Kastamonu Siyez bulgur, Kastamonu, Türkiye), Firik (Migros brand Firik bulgur, Hatay, Türkiye) and Karakilçik (Anatolian flavors Amik plain Karakilçik pilaf bulgur, Hatay, Türkiye) bulgur, non-iodized salt (Horoz brand, Denizli, Türkiye), and butter (Migros brand, Istanbul, Türkiye) were obtained from local markets in Denizli and drinking water available at the university was used for cooking (from February to May, 2024).

### 2.2 Preparation of bulgur pilaf

In this study, the amounts given in Table 1 of Siyez, Firik, and Karakilçik bulgurs (130–150 g), water (350–450 mL), butter (14 g), and salt (2 g) were used. Firstly, all ingredients except for bulgur were put into the pot for cooking the bulgur pilaf and then this mixing were heated until boiling (approximately 100 °C). Then, the bulgur was put in and cooked until it absorbed the water. The stove was switched off, and the cooked bulgur pilaf was steeped for 15 min. The prepared Siyez bulgur pilaf (SBP), Karakilçik bulgur pilaf (KBP) and Firik bulgur pilaf (FBP) were prepared ready for analyzing. While all chemical analyses were conducted in Food Technology Laboratory of Tekirdağ University, the sensory analyses were conducted in Pamukkale University.

**TABLE 1:** Design of an experiment to formulate bulgur pilafs using the response surface method.

Samples	Bulgur amount (g) (X_1_)	Water amount (mL) (X_2_)	Butter quantity (g)	Salt amount (g)
1	140 (0)	400 (0)	14	2
2	150 (+1.41)	400 (0)	14	2
3	140 (0)	400 (0)	14	2
4	140 (0)	400 (0)	14	2
5	135 (−1)	425 (+1)	14	2
6	145 (+1)	425 (+1)	14	2
7	145 (+1)	375 (−1)	14	2
8	140 (0)	350 (−1.41)	14	2
9	140 (0)	450 (+1.41)	14	2
10	140 (0)	400 (0)	14	2
11	135 (−1)	375 (−1)	14	2
12	140 (0)	400 (0)	14	2
13	130 (−1.41)	400 (0)	14	2

The simultaneous effects of bulgur and water quantities in the formulation of bulgur pilaf were investigated using response surface methodology. The Central Composite Design (CCD) method was used to design the study using the response surface method. The independent variables were provided, such as the amount of bulgur and water. Before creating the experimental design in the response surface method, the lower and upper limits of the independent variables used in the formulation were determined as bulgur amount 350–450 g and water amount 350–450 mL. These determined values were entered into the system, and the formulation trial design of bulgur pilafs was created according to the “Central Composite Design” method, which is used for the formulation of bulgur pilafs by the response surface method. The CCD matrix is given in Table 1.

### 2.3 Modeling procedure for response surface method (RSM)

Response surface method was preferred to examine the effects of bulgur (X_1_, 130–150 g) and water (X_2_, 350–450 mL) on selected response variables (taste, color, smell, and general acceptance). Minitab Statistical Analysis Software (MiniTable 18.1.1) was employed to determine the best, most liked bulgur pilafs based on the scoring of geographically marked Siyez, Firik and Karakilçik bulgur pilafs by sensory panelists. A central composite design was selected, and a five-level, two-factor [bulgur (g) and water (mL)] experimental design was created.

### 2.4 Sensory analysis

With some modifications, the described method in was applied to measure the sensory characteristics. Bulgur pilafs were tested for taste, color, smell, and general acceptance (overall acceptability) of the samples by 50 Semi-educated panelists (28 females, 22 males) at Pamukkale University Tavas Vocational School and Faculty of Tourism, Department of Gastronomy and Culinary Arts, aged between 18 and 50 years. All samples were presented randomly and coded using random three-digit numbers (29). The sensory testing environment was set up to prevent panelist effects. Panelists were informed about pretesting and testing procedures but not about bulgur varieties. The sensory analysis was tasted by panelists in groups of 25 people in two different sessions, at 10:00 in the morning and at 3:00 p.m. Samples were presented on white plates, allowing evaluation of colors and clarity in daylight. The sensory testing room’s temperature and humidity were 22 °C and 30%. After tasting the samples, water was offered to the panelists. Sensory characteristics were rated on a 9-point hedonic scale. Scale scores were reported as 9: excellent; 8: very good; 7: good; 6: acceptable 5: neither like nor dislike; 4: not bad/not good enough; 3: bad; 2: very bad; 1: terrible.

### 2.5 Total phenolic compounds (TPC)

The Folin-Ciocalteau method was used to quantify the TPC (30). The calibration curve in the spectrophotometer (SP-UV/VIS-300SRB, Spectrum Instruments, Melbourne, Australia) was created using gallic acid solutions in the range 5–100 mg/L concentration. TPC of the samples was analyzed following the Folin–Ciocalteu procedure with slight modifications. For extraction, 2 mL of sample was combined with 8 mL of methanol and centrifuged at 4000 rpm for 20 min. The obtained supernatant was mixed with Folin–Ciocalteu reagent, distilled water, and Na_2_CO_3_ solution. After a 2 h incubation period, absorbance values were recorded at 765 nm using a spectrophotometer. Quantification was carried out against a standard calibration curve prepared with gallic acid, and results were expressed as mg gallic acid equivalents (GAE) per 100 mL of sample.

### 2.6 Total flavonoid compounds (TFC)

The aluminum chloride colourimetric analysis method was used to determine the total flavonoid content (31). TFC was determined using the aluminum chloride colorimetric method. Briefly, 1 mL of sample was mixed with NaNO_2_ (5%), AlCl_3_ (10%), and NaOH (1 M), and the volume was adjusted to 10 mL with double-distilled water. After incubation at room temperature in the dark for 30 min, absorbance was measured at 510 nm using a UV–VIS spectrophotometer (SP-UV/VIS-300SRB, Spectrum Instruments, Melbourne, Australia). Quantification was performed using a catechin calibration curve, and results were expressed as mg catechin equivalents (CE)/L. All analyses were conducted in triplicate.

### 2.7 Antioxidant capacity determined by the DPPH assay

Since bulgur pilaf is a solid food matrix, an additional preparation step was required before antioxidant analysis. 1 g of homogenized bulgur pilaf was extracted with 10 mL of unacidified 80% methanol by vortexing at 1800 rpm for 15 min and storing at 4 °C for 18 h. Following extraction, the samples were centrifuged, and the clear liquid fraction (supernatant) obtained after sedimentation of solid particles was collected. DPPH radical scavenging activity was evaluated using the DPPH (2.2-diphenyl-1-picrylhydrazyl) method with the following changes (32). 0.1 mL of the supernatant was mixed with 2.9 mL of 0.1 mM DPPH solution in ethanol, vortexed, and incubated at room temperature for 30 min in the dark. The absorbance was read at 517 nm using a UV-VIS spectrophotometer (SP-UV/VIS-300SRB, Spectrum Instruments, Melbourne, Australia). The DPPH radical scavenging activity was calculated using Equation 1.


(1) $$DPPHradicalscavengingactivity\left(\%\right)=\left(A_{0}-A_{1}\right)/A_{0}\times 100$$

where A0 is the absorbance of the control and A1 is the absorbance of the bulgur pilaf.

### 2.8 Color parameter determination

A liquid beaker and a PCE-CSM 5 color meter were used to determine the color of the samples. The color was defined in terms of L (darkness-lightness), a (greenness-redness), and b (blue-yellowness) color parameters. The instrument was calibrated with a calibration plate before the measurements (33). Chroma (C) (34), which is used to measure color intensity, and h (hue angle) (35), which indicates the angle of the surface color, were calculated using the analysis data. C, h, and ΔE (total color change) were calculated using Equations 2.


(2) $$C=\left(a^{2}+b^{2}\right)\,1/2\;$$

$$h=t\,a\,n^{-1}\,\left(b/a\right)$$

$$△\,\text{E}=\left({\left(△\,L\right)}^{2}+{\left(△\,a\right)}^{2}+{\left(△\,b\right)}^{2}\right)\,1/2$$

### 2.9 Particle swarm optimization (PSO) algorithm

Stochastic optimization algorithms are often used in food technology to optimize complex, multivariable systems. Among these algorithms, PSO stands out as an effective tool, especially for solving multi-objective and global optimization problems (36). PSO has found a remarkable field of application in food technology due to its simple structure, rapid convergence and few adjustable parameters. The literature shows that PSO can deliver highly accurate results with less calculation time compared to other stochastic methods (37).

Particle swarm optimization is a stochastic optimization technique that iteratively updates randomly initialized particles to identify the optimal solution within a given search space. In this approach, each particle corresponds to a potential solution, while the collection of particles forms a swarm (population). The algorithm begins by randomly assigning the positions and velocities of the particles. Each particle moves based on both its own best position it has achieved in the past (personal best position, p_i_) and the best position in the entire swarm (global best position, p_g_). In each iteration, the velocities and positions of the particles are updated according to these two factors. The values of the objective function corresponding to the updated positions are calculated, and both the individual and global best values are updated. The process is repeated until the specified termination criterion (number of iterations) is reached and the optimal solution is obtained as a result of this cycle (38, 39). In each (t + 1) iteration, the position and velocity of the particles are calculated using the following Equations 3, 4:


(3) $$v_{i}\,\left(t+1\right)=w\,v_{i}\,\left(t\right)+c_{1}\,r_{1}\,\left(p_{i}\,\left(t\right)-x_{i}\,\left(t\right)\right)+c_{2}\,r_{2}\,\left(p_{g}\,\left(t\right)-x_{i}\,\left(t\right)\right)\,$$

(4) $$x_{i}\,\left(t+1\right)=x_{i}\,\left(t\right)+v_{i}\,\left(t+1\right)\;$$

During the iteration, each particle updates its velocity and position according to its best known position and the best global position found by the swarm. Where w is the inertia weight, *c*_1_ and *c*_2_ are the learning weights (weights of the cognitive and social components), and *r*_1_ and *r*_2_ are random numbers in the range (0.1).

### 2.10 Experimental design and hyperparameter tuning for PSO

The design of experiments and parameter optimization in connection with the PSO algorithm were carried out entirely using the Python programming language (version 3.11.3) in the Jupyter Notebook environment (version 6.5.4). All analyses, coding steps and visualization processes were carried out in an integrated manner in this interactive working environment.

The PSO hyperparameters were calibrated by successive trial and error. For each objective function, the swarm size was set to 10, the number of iterations to 40 and the cognitive (*c*_1_) and social (*c*_2_) coefficients to 0.10. The inertia coefficient w decreased linearly from 0.90 in the first iteration to 0.30. Each parameter set was evaluated in 30 independent runs due to the random nature; the best overall score, the average score and the standard deviation were reported.

### 2.11 Statistical analysis

The data obtained from the analyses with bulgur pilafs were evaluated by one-way analysis of variance (ANOVA), and the differences between the means were determined using the Tukey HSD test at *p* < 0.05 significance level. Statistical analysis was performed using SPSS 22.0 software (SPSS Inc., Chicago, USA). Before applying ANOVA, the normality of the data was checked with the Shapiro-Wilk test (*p* > 0.05), and Q-Q plots were examined to verify the normality of the residuals. Since the normality assumption was met, parametric tests were applied. Each experimental condition was repeated three times (*n* = 3), and mean values and standard deviations (SD) for each experimental point were calculated and reported. Principal Component Analysis (PCA) was performed using JMP software (version 12.2.0, SAS Institute, Cary, NC, USA) to examine the relationships between variables and to visualize the grouping of different bulgur pilaf types (Siyez, Firik, Karakılçık).

## 3 Results and discussion

### 3.1 Optimization of sensory parameters

Bulgur is a whole wheat product produced mainly from durum wheat (*Triticum durum*) by cooking, drying, bran separation, grinding, and size classification. Siyez bulgur (*Triticum monoccoccum* L.), one of the bulgur varieties with geographical indication, is a well-established traditional food consumed in some parts of Türkiye since ages (40). Hulled wheat such as Siyez bulgur are essential for the local economy in Türkiye due to their higher yield and easier processing than *T. durum* (41). Bulgur, a semi-processed product, can be cooked quickly and is easily digested. Bulgur, which has a neutral taste, is included in many food recipes such as soup, meatballs, dessert, and pilaf because it is compatible with many flavors such as salty, sweet, sour, and bitter. By grinding in different sizes, bulgur is obtained in various sizes, expanding the network of use of bulgur in meals (8).

For optimization, 13 trial points were determined (Table 1). Model adequacy, R^2^, adjusted-R^2^ coefficients, lack-of-fit tests, and analysis of variance (ANOVA) were used to evaluate the statistical significance of the model (*p* < 0.05). The experimental design of Siyez bulgur pilaf in Table 2, Firik bulgur pilaf in Table 3 and Karakilçik bulgur pilaf in Table 4 are presented. To create the equation models, the following second-order polynomial Equation 5 was used:


(5) y=b0+b1⁢X1+b2⁢X2⁢b12⁢X1⁢X2+b11⁢X12+b22⁢X22  

**TABLE 2:** Siyez bulgur pilaf RSM measured responses used in experimental design.

Run no	Independent variables		Dependent variables							
	Karakilçik bulgur (X_1_) (g)	Water (X_2_) (mL)	Taste		Color		Smell		General acceptance	
			Experimental data	RSM predicted	Experimental data	RSM predicted	Experimental data	RSM predicted	Experimental data	RSM predicted
140	400	7.15 ± 0.07	7.25	6.67 ± 0.72	6.65	5.40 ± 0.38	5.46	6.79 ± 0.06	6.80	140
150	400	6.99 ± 0.11	7.10	7.51 ± 0.61	7.49	7.33 ± 0.06	7.45	6.64 ± 0.89	6.65	150
140	400	7.15 ± 0.20	7.25	6.67 ± 0.61	6.65	5.40 ± 0.21	5.46	6.79 ± 0.30	6.80	140
140	400	7.15 ± 0.04	7.25	6.67 ± 0.74	6.65	5.40 ± 0.27	5.46	6.79 ± 0.47	6.80	140
135	425	6.65 ± 0.08	6.63	7.17 ± 0.08	7.04	5.5 ± 0.14	5.54	6.32 ± 0.93	6.21	135
145	425	7.56 ± 0.13	7.54	7.00 ± 0.52	6.90	6.34 ± 0.16	6.27	7.18 ± 0.07	7.08	145
145	375	6.52 ± 0.20	6.68	6.94 ± 0.23	6.97	6.7 ± 0.07	6.79	6.19 ± 0.79	6.26	145
140	350	6.28 ± 0.11	6.29	6.43 ± 0.20	6.36	6.8 ± 0.62	6.78	5.97 ± 0.55	5.89	140
140	450	6.60 ± 0.07	6.80	6.97 ± 0.25	7.01	6.36 ± 0.28	6.51	6.27 ± 0.75	6.37	140
140	400	7.15 ± 0.27	7.25	6.67 ± 0.14	6.65	5.40 ± 0.54	5.46	6.79 ± 0.13	6.80	140
135	375	6.82 ± 0.11	6.98	6.31 ± 0.61	6.32	5.10 ± 0.31	5.30	6.48 ± 0.18	6.54	135
140	400	7.15 ± 0.16	7.25	6.67 ± 0.34	6.65	5.40 ± 0.20	5.46	6.79 ± 0.13	6.80	140
130	400	6.37 ± 0.08	6.47	6.99 ± 0.27	6.98	5.23 ± 0.18	5.23	6.05 ± 1.27	6.06	130
150	450	7.75	7.08		7.67		7.37			
Experimental values			7.50 ± 0.31		7.19 ± 0.06		7.83 ± 0.38		7.75 ± 0.27	
% difference			3.22		1.52		2.04		4.90	

X_1_, Siyez bulgur; X_2_, water; RSM, response surface methodology; S-BP, Siyez bulgur pilaf.

**TABLE 3:** Firik bulgur pilaf RSM measured responses used in experimental design.

Run no	Independent variables		Dependent variables							
	Karakilçik bulgur (X_1_) (g)	Water (X_2_) (mL)	Taste		Color		Smell		General acceptance	
			Experimental data	RSM predicted	Experimental data	RSM predicted	Experimental data	RSM predicted	Experimental data	RSM predicted
1	140	400	7.51 ± 0.45	7.51	6.84 ± 0.23	6.78	5.67 ± 0.10	5.59	6.07 ± 0.13	6.01
2	150	400	6.53 ± 0.07	6.57	7.42 ± 0.19	7.36	7.68 ± 0.56	7.67	6.29 ± 0.03	6.19
3	140	400	7.51 ± 0.14	7.51	6.84 ± 0.37	6.78	5.67 ± 0.17	5.59	6.07 ± 0.17	6.01
4	140	400	7.51 ± 0.18	7.51	6.84 ± 0.49	6.78	5.67 ± 0.11	5.59	6.07 ± 0.15	6.01
5	135	425	6.74 ± 0.21	6.85	6.96 ± 0.25	7.03	5.43 ± 0.24	5.57	5.53 ± 0.35	5.60
6	145	425	7.15 ± 0.16	7.17	7.2 ± 0.06	7.24	7.54 ± 0.14	7.52	7.46 ± 0.19	7.57
7	145	375	7.01 ± 0.24	7.00	6.33 ± 0.46	6.36	5.98 ± 0.58	5.94	4.8 ± 0.34	4.91
8	140	350	6.84 ± 0.40	6.83	5.8 ± 0.21	5.74	6.07 ± 0.10	6.00	6.11 ± 0.10	6.03
9	140	450	6.60 ± 0.07	6.61	7.04 ± 0.14	6.96	7.81 ± 0.07	7.70	7.51 ± 0.41	7.45
10	140	400	7.51 ± 0.22	7.51	6.84 ± 0.38	6.78	5.67 ± 0.16	5.59	6.07 ± 0.58	6.01
11	135	375	7.16 ± 0.31	7.24	6.63 ± 0.16	6.68	5.36 ± 0.05	5.46	6.78 ± 0.12	6.85
12	140	400	7.51 ± 0.25	7.51	6.84 ± 0.30	6.78	5.67 ± 0.18	5.59	6.07 ± 0.29	6.01
13	130	400	6.53 ± 0.20	6.49	7.54 ± 0.37	7.46	5.4 ± 0.06	5.24	6.22 ± 0.09	6.17
S-BP	147	435	6.68		7.52		8.7		8.75	
Experimental values			7.12 ± 0.23		7.08 ± 0.21		8.46 ± 0.41		8.49 ± 0.14	
% difference			6.17		5.85		2.75		2.97	

X_1_, Firik bulgur; X_2_, water; RSM, response surface methodology; F-BP, Firik bulgur pilaf.

**TABLE 4:** Karakilçik bulgur pilaf RSM measured responses used in experimental design.

Run no	Independent variables		Dependent variables							
	Karakilçik bulgur (X_1_) (g)	Water (X_2_) (mL)	Taste		Color		Smell		General acceptance	
			Experimental data	RSM predicted	Experimental data	RSM predicted	Experimental data	RSM predicted	Experimental data	RSM predicted
1	140	400	6.53 ± 0.07	6.61	7.25 ± 0.10	7.12	6.72 ± 0.03	6.75	7.15 ± 0.57	7.09
2	150	400	5.68 ± 0.16	5.77	6.73 ± 0.49	6.62	6.69 ± 0.11	6.70	6.25 ± 0.03	6.17
3	140	400	6.53 ± 0.25	6.61	7.25 ± 0.21	7.12	6.72 ± 0.31	6.75	7.15 ± 0.21	7.09
4	140	400	6.53 ± 0.27	6.61	7.25 ± 0.08	7.12	6.72 ± 0.23	6.75	7.15 ± 0.27	7.09
5	135	425	6.43 ± 0.10	6.54	6.84 ± 0.18	6.83	6.16 ± 0.79	6.22	6.73 ± 0.35	6.74
6	145	425	6.76 ± 0.15	6.85	7.48 ± 0.20	7.42	7.58 ± 0.11	7.67	7.44 ± 0.44	7.45
7	145	375	5.83 ± 0.04	5.88	6.6 ± 0.14	6.54	5.85 ± 0.05	5.90	6.37 ± 0.54	6.39
8	140	350	5.85 ± 0.24	5.92	5.97 ± 0.16	5.85	5.85 ± 0.73	5.88	6.02 ± 0.20	5.95
9	140	450	7.05 ± 0.35	7.13	7.47 ± 0.07	7.32	7.23 ± 0.95	7.26	7.75 ± 0.07	7.68
10	140	400	6.53 ± 0.04	6.61	7.25 ± 0.16	7.12	6.72 ± 0.08	6.75	7.15 ± 0.18	7.09
11	135	375	6.23 ± 0.31	6.30	6.25 ± 0.69	6.24	6.6 ± 0.17	6.62	6.06 ± 0.41	6.07
12	140	400	6.53 ± 0.10	6.61	7.25 ± 0.28	7.12	6.72 ± 0.14	6.75	7.15 ± 0.35	7.09
13	130	400	5.82 ± 0.19	5.88	5.88 ± 0.06	5.73	5.92 ± 0.21	5.97	5.21 ± 0.48	5.15
S-BP	146	450	7.16		7.54		8.58		7.76	
Experimental values			7.23 ± 0.07		7.68 ± 0.07		8.45 ± 0.42		7.92 ± 0.1	
% difference			0.96		1.82		1.51		2.02	

X_1_, Karakilçik bulgur; X_2_, water; RSM, response surface methodology; K-BP, Karakilçik bulgur pilaf.

The coefficients of the polynomial are represented by b_0_ (constant term), b_1_ and b_2_ (linear effects), b_11_ and b_22_ (quadratic effects), and b_12_ (interaction effects).

Response surface method is a mathematical method for obtaining statistically acceptable results (33). It allows the evaluation of multiple parameter effects on response variables and predicts behavior under specific conditions (42). RSM modeling was used to obtain the Siyez bulgur pilaf most liked by consumers. The effect of the independent variables on the taste of Siyez bulgur pilaf is shown by the result of mathematical Equation 6 of the regression analysis.


(6) $$T\,a\,s\,t\,e=-171.9+2.298\,X_{1}-0.1073\,X_{2}-0.004678\,X_{1}\,X_{1}$$

$$-0.000283\,X_{2}\,X_{2}+0.002420\,X_{1}\,X_{2}\;$$

According to the equation, the increase in the amount of X_1_ (g) positively affected the taste result of Siyez bulgur pilaf by showing a linear effect. In contrast, the increase in the amount of X_2_ (mL) affected it negatively. It was noted that the taste result of bulgur pilaf was negatively affected by the quadratic effects of these independent variables and positively affected by the interaction effects. The impact of independent factors on the color of Siyez bulgur pilaf was obtained by the mathematical Equation 7 from regression analysis. The regression analysis results are presented in Equations 8, 9 showing the effect on smell and general acceptance.


(7) $$C\,o\,l\,o\,r=28.3-0.977\,X_{1}+0.2183\,X_{2}+0.005856\,X_{1}\,X_{1}$$

$$+0.000014\,X_{2}\,X_{2}-0.001593\,X_{1}\,X_{2}\;$$

(8) $$S\,m\,e\,l\,l=153.8-1.744\,X_{1}-0.1675\,X_{2}+0.008797\,X_{1}\,X_{1}$$

$$+0.000472\,X_{2}\,X_{2}-0.001520\,X_{1}\,X_{2}\;$$

(9) $$G\,e\,n\,e\,r\,a\,l\,A\,c\,c\,e\,p\,t\,a\,n\,c\,e=-0.6+0.354\,X_{1}-0.1019\,X_{2}$$

$$-0.004444\,X_{1}\,X_{2}-0.000269\,X_{2}\,X_{2}+0.002299\,X_{1}\,X_{2}\;$$

According to the equations, it was seen that the color and smell findings of Siyez bulgur pilaf were positively affected by the squared effects of X_1_ and X_2_ variables. It was determined that the general acceptance finding of Siyez bulgur pilaf was positively affected by the interaction effects of X_1_ and X_2_ variables.

Analysis of variance (ANOVA) results for taste, color, smell, and general acceptance values of Siyez bulgur pilaf are presented in Supplementary Table 1. ANOVA results were significant (*p* < 0.01), and a high coefficient of determination (R^2^) was determined. The acceptability of Siyez bulgur pilaf was influenced by X_1_ and X_2_ (*p* < 0.01). The quadratic effect of X_1_ significantly affected Siyez bulgur pilaf taste, color, smell and general acceptance scores (*p* < 0.01). The square effect of X_2_ had no significant impact on pilaf color (*p* > 0.05). In contrast, it showed a statistically significant impact on taste, smell, and general acceptance (*p* < 0.01). The R^2^ values showed high agreement with 96.90%, 97.77%, 99.10% and 99.10% for taste, color, smell and general acceptance, respectively (Supplementary Table 1). Similar to our study, Aydoğdu et al. (43) optimized sensory attributes using RSM. They found that R^2^ values showed high agreement with 98.23% for color, 99.40% for smell, 98.44% for taste, and 99.75% for general acceptance (43).

**FIGURE 2:**
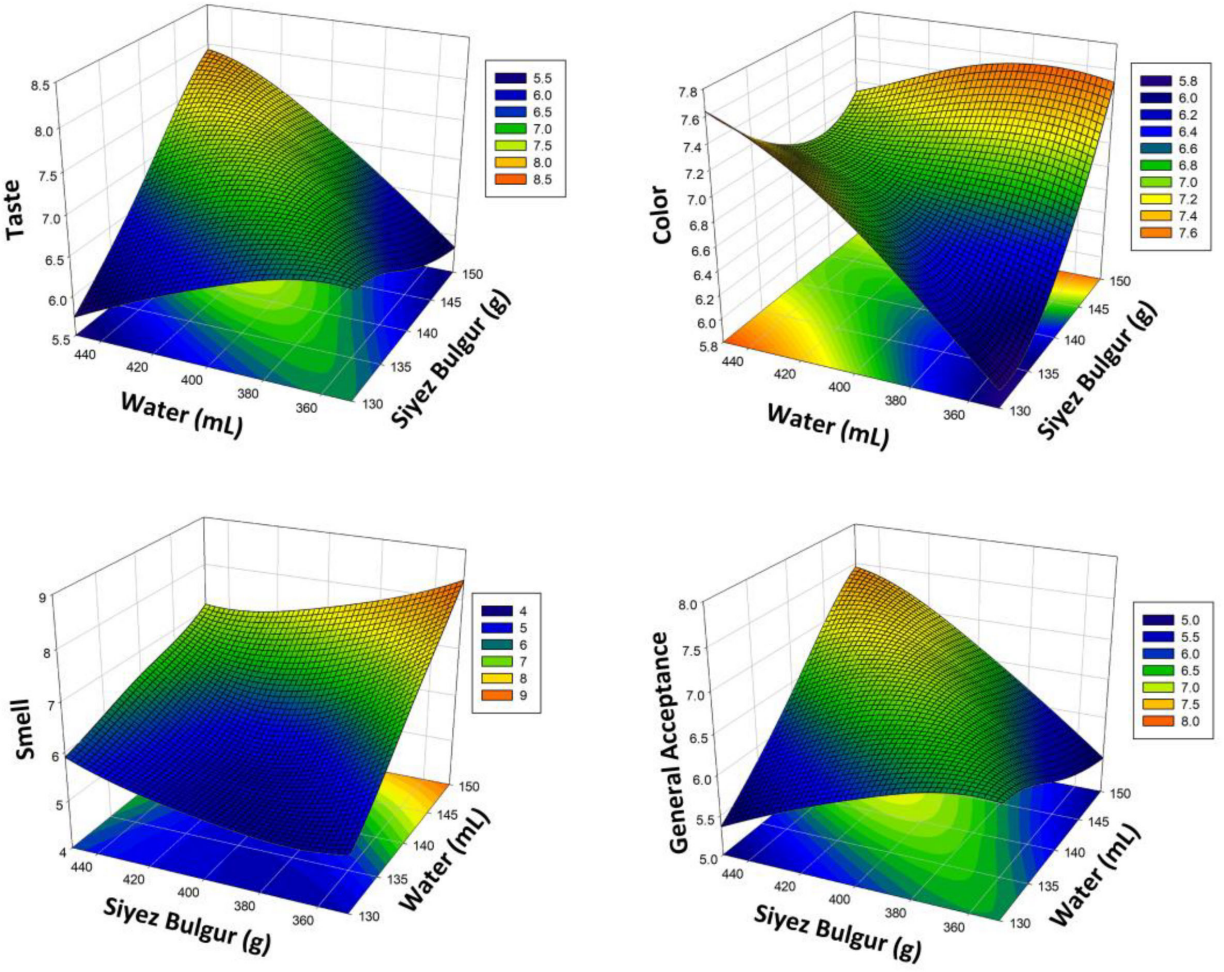
Response surface plots (3D) for taste, color, smell, and general acceptance analysis as a function of significant interaction factors for RSM (Siyez bulgur pilaf).

The Siyez bulgur pilaf values obtained by using RSM and the effect of variables X_1_ and X_2_ on taste, color, smell, and general acceptance with the values obtained by repeated measurements were explained by 3D graphs and linear regressions shown in Figure 2. The optimal levels of the independent variables on taste, color, smell, and general acceptance were determined (150 g of bulgur and 450 mL of water). Under these conditions, the taste score was calculated as 7.75, the color score as 7.08, the smell score as 7.67, and the general acceptance as 7.37. While the figure was used to find the optimum cooking conditions, it was observed that the taste and general acceptance scores increased with the amount of water. In particular, while the increase in the amount of water had a positive effect up to a certain point, a decrease in the taste and general acceptance scores was observed when this amount exceeded the optimum level. This situation reveals that the controlled adjustment of the amount of water is critical to obtaining the desired quality.

R^2^ values, ANOVA, incompatibility, and regression coefficients of the geographically marked Firik bulgur pilaf are shown in Supplementary Table 2. RSM modeling was used to obtain the Firik bulgur pilaf that is most liked by consumers. The second-order modeling equations for the sensory parameter values of taste, color, smell and general acceptance of Firik bulgur pilaf are presented as Equations 10–13, respectively, as a result of the optimization.


(10) $$T\,a\,s\,t\,e=-171.9+2.298\,X_{1}+0.0935\,X_{2}-0.009795\,X_{1}\,X_{1}$$

$$-0.000316\,X_{2}\,X_{2}+0.001122\,X_{1}\,X_{2}\;$$

(11) $$C\,o\,l\,o\,r=159.0-2.200\,{\text{X}}_{1}-0.0015\,{\text{X}}_{2}+0.006303\,{\text{X}}_{1}\,{\text{X}}_{1}$$

$$-0.000171\,{\text{X}}_{2}\,{\text{X}}_{2}+0.001075\,{\text{X}}_{1}\,{\text{X}}_{2}\;$$

(12) $$S\,m\,e\,l\,l=397.6-3.481\,X_{1}-0.8012\,X_{2}+0.008632\,X_{1}\,X_{1}$$

$$+0.000504\,X_{2}\,X_{2}+0.002964\,X_{1}\,X_{2}\;$$

(13) $$G\,e\,n\,e\,r\,a\,l\,A\,c\,c\,e\,p\,t\,a\,n\,c\,e=519.4-3.620\,X_{1}-1.3146\,X_{2}$$

$$+0.001766\,X_{1}\,X_{1}+0.000293\,X_{2}\,X_{2}+0.007817\,X_{1}\,X_{2}\;$$

The equations showed that X_1_ (g) increased positively affected the taste of Firik bulgur pilaf, and negatively affected color, smell and general acceptance. The X_2_ increase (mL) had a negative effect on color, smell and general acceptance, but a positive effect on taste. Bulgur pilaf was found to be affected by the effects of the independent variables and the interaction effects. The color parameter was positively influenced by X_1_ and negatively by X_*dfc*_, and positively by the interaction of the two. The interaction and quadratic effects of the independent variables positively influenced smell and acceptability.

**FIGURE 3:**
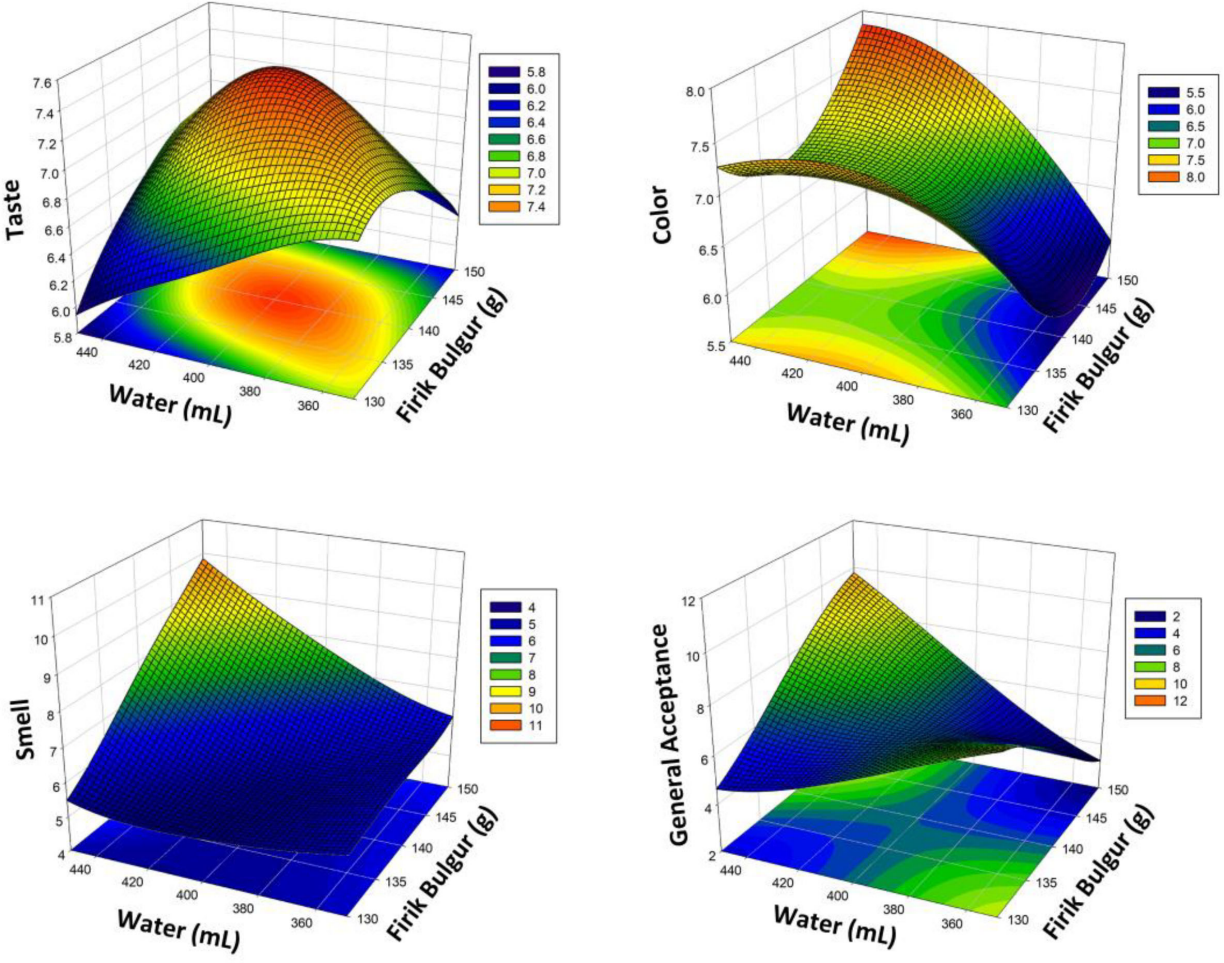
Response surface plots (3D) for taste, color, smell, and general acceptance analysis as a function of significant interaction factors for RSM (Firik bulgur pilaf).

Supplementary Table 2 shows the results of an ANOVA of sensory attributes of Firik bulgur pilaf. X_1_ and X_2_ had a significant effect on taste, color, smell and general acceptance (*p* < 0.05). The cross-interactions of factors X_1_ and X_2_ on bulgur pilaf are statistically significant for taste, color and smell (*p* < 0.05). Cross-interaction of factor X_1_ is not statistically significant for general acceptance (*p* > 0.05). RSM modeling has a high fit (99.05%, 98.40%, 99.17% and 98.86%, respectively). Two-way and one-way effects of the modeling are statistically significant (*p* < 0.05). Figure 3 shows the response surface plot explaining the effect of variables X_1_ and X_2_ on taste, color, smell and general acceptance. The optimal levels of the independent variables on taste, color, smell, and general acceptance were determined (147 g of bulgur and 435 mL of water). Under these conditions, the taste score is 6.68, the color score is 7.52, the smell score is 8.7, and the general acceptance is 8.75. The figure shows that the taste and general acceptance variables change in direct proportion to the amount of water. In particular, the increase in the amount of water positively affects the taste quality up to a certain point. In contrast, excessive water use can negatively affect the taste and general acceptance scores. These findings show that the optimum cooking conditions in Firik bulgur pilaf depend on the balanced use of the amount of water.

The Karakilçik bulgur pilaf was determined using RSM modeling based on the geographical indication that consumers liked it the most. Equations 14–17 show the second-order model equations for the sensory parameters of the taste, color, smell and general acceptance of Karakilçik bulgur pilaf, based on the optimization.


(14) $$T\,a\,s\,t\,e=-73.69+1.5937\,X_{1}-0.16647\,X_{2}-0.007803\,X_{1}\,X_{1}$$

$$-0.000033\,X_{2}\,X_{2}+0.001464\,X_{1}\,X_{2}\;$$

(15) $$C\,o\,l\,o\,r=-193.2+2.476\,X_{1}+0.1047\,X_{2}-0.009512\,X_{1}\,X_{1}$$

$$-0.000214\,X_{2}\,X_{2}+0.000580\,X_{1}\,X_{2}\;$$

(16) $$S\,m\,e\,l\,l=145.54-0.5278\,X_{1}-0.5358\,X_{2}-0.004189\,X_{1}\,X_{1}$$

$$-0.000073\,X_{2}\,X_{2}+0.004343\,X_{1}\,X_{2}\;$$

(17) $$G\,e\,n\,e\,r\,a\,l\,A\,c\,c\,e\,p\,t\,a\,n\,c\,e=-259.5+3.727\,{\text{X}}_{1}-0.0068\,{\text{X}}_{2}$$

$$-0.014254\,{\text{X}}_{1}\,{\text{X}}_{1}-0.000108\,{\text{X}}_{2}\,{\text{X}}_{2}+0.000789\,{\text{X}}_{1}\,{\text{X}}_{2}\;$$

According to the equations, it was determined that the increase in the amount of X_1_ (g) positively affected the taste, color and general acceptance parameters of Karakilçik bulgur pilaf by showing a linear effect. The quadratic effects of X_1_ and X_2_ negatively affected the taste, color, smell and general acceptance parameters of Karakilçik bulgur pilaf.

The ANOVA results of the sensory properties of taste, color, smell, and general acceptance of Karakilçik bulgur pilaf are given in Supplementary Table 3. The linear effects of taste, color, smell, and general acceptance of X_1_ and X_2_ on Karakilçik bulgur pilaf are significant (*p* < 0.05). The square interactions of X_1_ and X_2_ factors on bulgur pilaf were statistically significant for the sensory parameters of taste, color, smell, and general acceptance (*p* < 0.05). High agreement was shown by the R^2^ values of the RSM modeling level with 99.93%, 99.32%, 99.86%, and 99.72%, respectively.

**FIGURE 4:**
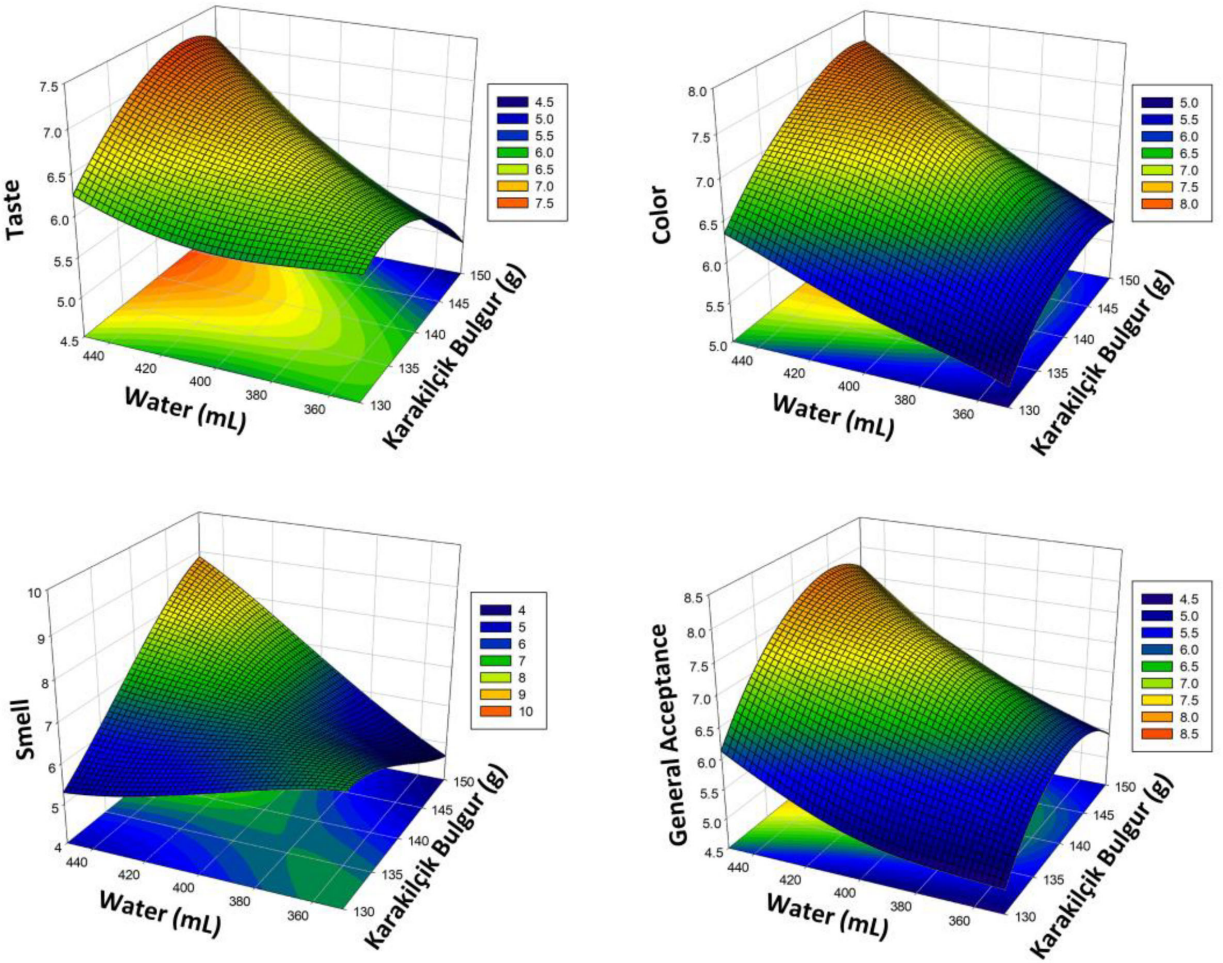
Response surface plots (3D) for taste, color, smell, and general acceptance analysis, showing the effect of significant interaction factors on RSM (Karakilçik bulgur pilaf).

The Karakilçik bulgur pilaf values obtained using RSM and the effects of X_1_ and X_2_ variables on taste, color, smell, and general acceptance are presented with 3D graphics in Figure 4. The optimal levels of independent variables on taste, color, smell, and general acceptance were determined (146 g of bulgur and 450 mL of water). Under these conditions, the taste score was calculated as 7.16, the color score as 7.54, the smell score as 8.58, and the general acceptance as 7.76. This figure clearly shows how the taste and general acceptance parameters change depending on the amount of water and the bulgur ratio. The results show that carefully adjusting the amount of water for Karakilçik bulgur significantly affects taste and general acceptance. Significant differences in parameters such as color and smell were also observed depending on the amount of water and bulgur used. The graph provides valuable information for determining the optimal cooking conditions.

Differences in bulgur and water content appear to lead to changes in the sensory and nutritional properties of the product. A higher bulgur content increases the degree of starch gelatinization, resulting in higher textural density, which consumers perceive as a fuller mouthfeel. Similarly, an increase in water level facilitates the extraction of phenolic compounds; however, excessive water dilutes color intensity and can reduce overall acceptance. These results suggest that the observed differences are due to physicochemical interactions occurring within the food matrix.

### 3.2 Bioactive results of bulgur pilafs

**FIGURE 5:**
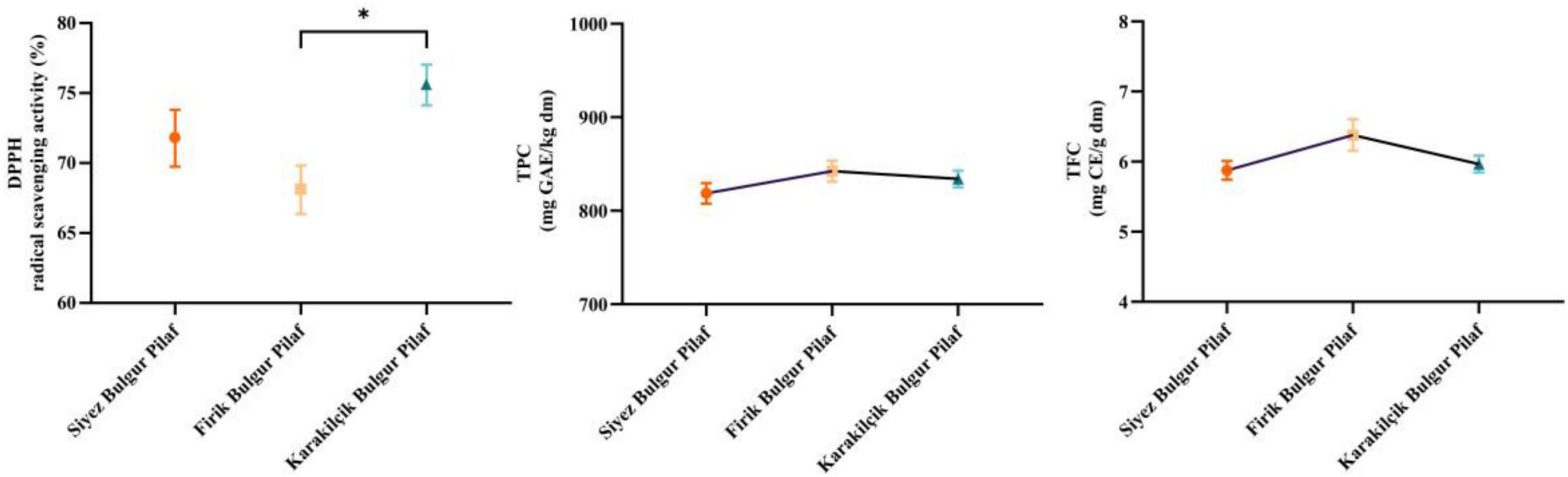
DPPH radical scavenging activity (DPPH) (%), total phenolic compounds (TPC) and total flavonoid compounds (TFC) results of bulgur pilafs. The symbols at the top of the bars indicate statistically significant differences (*p < 0.05).

In this study, the bioactive properties of Siyez Bulgur Pilaf (SBP), Firik bulgur pilaf (FBP), and Karakilçik bulgur pilaf (KBP) were compared in terms of DPPH radical scavenging activity, TPC, and TFC. Figure compares these bioactive properties (Figure 5). According to DPPH (% inhibition) results (Figure 5), Karakilçik bulgur pilaf (KBP) had the highest antioxidant capacity with 75.575. At the same time, Siyez bulgur pilaf (SBP) showed 71.78 and Firik bulgur pilaf (FBP) showed lower capacities with 68.1 values. The DPPH difference between SBP and FBP was 5.42%, between SBP and KBP was 5.02%, and between FBP and KBP was 9.88%. Statistical analyses showed that the difference between FBP and KBP was significant (*p* < 0.05), while the differences between SBP and other species were insignificant. These findings suggest that KBP’s antioxidant activity is statistically significantly higher than that of FBP, but not significantly different to that of SBP. While the differences in wheat varieties affect the antioxidant activity results of bulgur pilafs, short cooking time and low drying temperature in the bulgur process provide better preservation of the antioxidant activity values of bulgur (18). In the study, Ertop (44) reported that the antioxidant activity properties of Siyez, bulgur, and flour obtained from Siyez may vary depending on the processing and raw material applied. The antioxidant activity values of handmade and industrially obtained Siyez bulgur were determined as 23.10% and 24.79%, respectively (44). The DPPH value of Siyez wheat was determined to be 28.26% (45). The study conducted by Alkan (46) determined that the antioxidant activities of the local black clay varieties and genotypes used varied between 14.7 and 40.2 μmol TE/g (46).

In terms of total phenolic substance content (TPC) (Figure 5), Firik bulgur pilaf (FBP) had the highest value with 842.395 mg GAE/kg dm, followed by Karakilçik bulgur pilaf (KBP) with 834.005 mg GAE/kg and Siyez bulgur pilaf (SBP) with 818.695 mg GAE/kg values. The difference in TPC between SBP and FBP was calculated as 2.81%, the difference between SBP and KBP was 1.84%, and the difference between FBP and KBP was 1.01%. However, these differences were not statistically significant, i.e., no significant difference between bulgur pilafs in total phenolic substance content. This shows that although FBP showed the highest value in phenolic substance content, it did not create a statistically significant difference with the other types.

Pekkirişçi (47) and Pekkirişçi et al. (18) found that the TPC values of Siyez and Firik bulgur were 5327.15 and 7432.71 mg GAE/kg, respectively, and firik samples contained higher TPC (18, 47). Firik samples were reported to have higher levels of TPC. This was because they were obtained from wheat without physiological maturity. In addition, differences between wheat varieties, different cooking and drying parameters during bulgurization, and other processes such as roasting, milling, and bran separation effectively found various levels of phenolics (45, 47).

In a study in which the total phenolic matter contents of Siyez bulgur with and without husks were examined by cooking for 0–4 h, it was determined that the total phenolic matter content of the husked bulgur (3502.8–4301.9 mg kg^–1^ in dry matter) had higher average values than the one with husks removed (914.9–1014.9 mg kg^–1^ in dry matter) due to the high contribution of the husks; it was determined that the total phenolic matter content increased with increasing cooking time (40). The total phenolic content of Siyez bulgur pilaf was determined to be 1694.79 μg g^–1^ (GAE) in dry matter by Yilmaz and Koca (45).

In the study conducted by Zengin (15), it was determined that the antioxidant activity of Siyez wheat varied between 16.78% and 26.18%; the average value was 20.14%; the amount of total phenolic matter was between 1.47 and 1.63 mg GAE/g and the average was 1.54 mg GAE/g (15). Can (48) determined that the DPPH radical scavenging activity values of Siyez wheats taken from 3 different locations varied between 363.87 and 474.00 μg trolox equivalent/mg, and total phenolic matter contents varied between 363.81 and 401.91 mg GAE/kg (48).

In terms of total flavonoid content (TFC) (Figure 5C), Firik bulgur pilaf (FBP) had the highest value with 6.38 mg CE/g dm. In comparison, Karakilçik bulgur pilaf (KBP) showed lower flavonoid content with 5.965 mg CE/g dm and Siyez bulgur pilaf (SBP) with 5.875 mg CE/g dm. The difference in TFC between SBP and FBP was 7.91%, between SBP and KBP was 1.51%, and between FBP and KBP was 6.97%. There were no statistically significant differences in TFC values or flavonoid content between bulgur pilafs. This shows that although FBP has a high flavonoid content value, it does not create a statistically significant difference from other types.

As a result, Karakilçik bulgur pilaf (KBP) has the highest antioxidant capacity, while Firik bulgur pilaf (FBP) has the highest values in terms of both total phenolic and flavonoid content. However, according to DPPH results, the difference between FBP and KBP was statistically significant (*p* < 0.05). These findings provide vital information to determine how bulgur pilafs differ in bioactive component contents and the potential health benefits of these differences. High levels of bioactive components increase the functional properties of these foods and show the potential to contribute to consumer health.

The findings demonstrate that both nutritional value and consumer acceptance can be optimized in bulgur pilaf production. The positive correlation between total phenolic content and antioxidant activity, in particular, and overall acceptance presents a significant opportunity for functional food development. Furthermore, determining optimal bulgur-to-water ratios can help ensure consistent quality across a variety of applications, from home consumption to industrial production. In this respect, the study results offer concrete recommendations for both improving consumer satisfaction and developing health-supporting products.

### 3.3 Color values of bulgur pilafs

**FIGURE 6:**
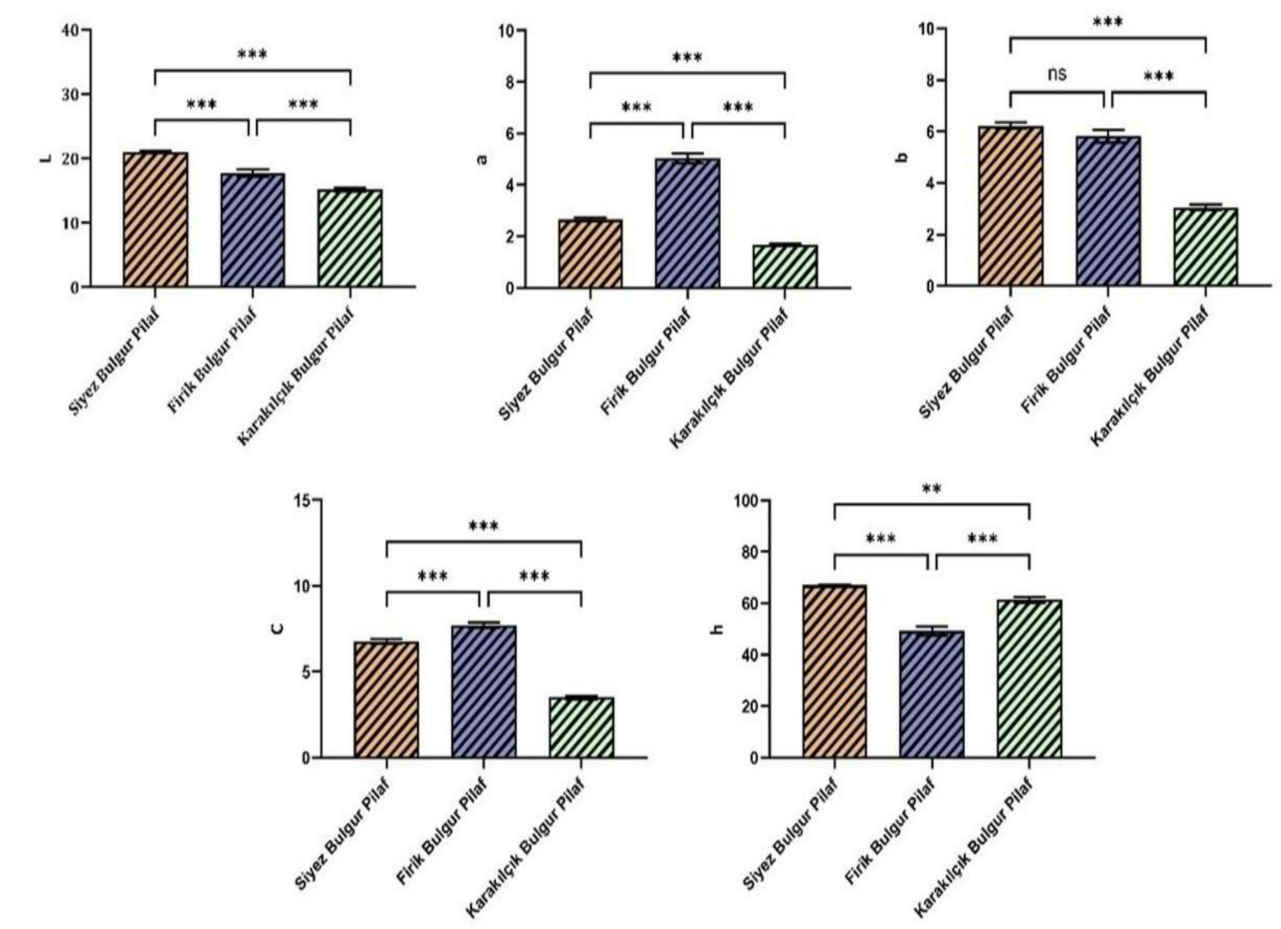
Color analysis results of bulgur pilafs. Letters at the top of the bars indicate statistically significant differences (*n* = 3 ± SD). Statistically significant differences are indicated by the symbols at the top of the bars (**p < 0.01, ***p < 0.001) and represent the effects observed under fasting conditions. ns: not significant differences. ns, not significant.

The color analysis results of bulgur pilafs obtained from geographically marked bulgur varieties are given in Figure 6. When the L and b values in Figure 6 are analyzed (Figures 6A, C), Siyez bulgur pilaf had the highest L (21.03) and b (6.23) values, while Karakilçik had the lowest L (15.15) and b (3.06) values. In other words, Siyez bulgur is lighter in color and more yellow than the others, while Karakilçik bulgur pilaf has the darkest color and the least yellow. Pekkirişçi (47) stated in his study that there was no statistical difference between the average L* values (50.48 and 49.85) of cooked Firik and Siyez bulgur. The fact that the raw materials and production methods used in bulgur production are very different from each other causes different brightness values to be obtained among bulgur varieties (47). It has been reported that the formation of yellow color in wheat and bulgur is caused by lutein, a carotenoid (40, 47, 49). It is reported that Siyez bulgur is dark yellow (45). In this case, in our study, it is thought that Siyez bulgur has more yellowness due to its higher lutein content, and Karakilçik bulgur, which contains less lutein, has less yellowness. On average, the total amount of yellow pigment calculated as the lutein equivalent value was found to be 5.4 times higher in the firik samples than in the Siyez samples (47).

Firik bulgur pilaf had the highest value (5.03), followed by Siyez bulgur pilaf (2.65) and then Karakilçik bulgur pilaf (1.67) in the redness value that is a value (Figure 6B). The possibility of freekeh wheat being harvested at different maturation times directly affects the redness/greenness value. The Maillard reaction, which may occur during uncontrolled drying or roasting processes, can cause some bulgur samples to appear redder. In addition, different particle sizes in bulgur also affect the redness value. As the particle size decreases, the light refraction is multi-directional, which leads to a decrease in the redness value (47, 50–52). Reported in his study examining the effect of particle size on color in wheat that particle size has a significant impact in color values, and that as particle size increases, there is a decrease in the L value and an increase in the a value. It has also been reported that bulgur’s redness (a) and yellowness (b) values can be affected by its drying methods (53). Yilmaz and Koca found the L, a, and b values of cooked Siyez bulgur to be 38.87, 7.13, and 15.23 on average, respectively, and compared them with bulgur obtained from durum wheat (45). It was determined that Siyez bulgur was less bright, more red and less yellow than durum wheat. The C (chroma) value, which expresses the tone of the color, determines whether it is vivid or pale (54). The highest C value, Firik bulgur pilaf (7.69), was observed to be more vibrant in color. In contrast, the lowest C value, Karakilçik bulgur pilaf (3.49), was observed to be paler in color than the others (Figure 6D). When the hue angle (h°) values were examined, they were listed from highest to lowest as Siyez bulgur pilaf (66.94), Karakilçik bulgur pilaf (61.31) and Firik bulgur pilaf (49.18) (Figure 6E). While the high hue angle values indicate that the samples have more intense colors, the color intensity decreases as these values decrease (55). This shows that the higher the Hue angle (H) and chroma (C) values, the better they represent the consumer’s color perception (56). Pekkirişçi (47) reported in her study that the average Hue value of cooked Firik bulgur (88.49) was higher than

### 3.4 Sensory analysis results of bulgur pilafs

This study investigated the sensory properties of SBP, FBP, and KBP bulgur pilafs comparatively. Sensory analysis results reveal the differences between these bulgur pilafs regarding taste, color, smell, and general acceptance. In taste evaluation, SBP bulgur pilaf had the highest score with 7.5, while FBP had 7.12 and KBP had 7.23 scores. These results show no statistically significant difference in taste (*p* > 0.05). In color evaluation, KBP bulgur pilaf had the highest score with 7.68, while SBP had 7.19 and FBP had 7.08 scores. There was a statistically significant difference between the bulgur pilafs in terms of color (*p* < 0.05). In the smell test, KBP got the highest score of 8.58, FBP got 8.46, and SBP got 7.67. There was also no statistically significant difference in smell (*p* > 0.05). Regarding general acceptance, FBP bulgur pilaf has the highest score with 8.49, while KBP has 7.92 and SBP has 7.35 scores. In general acceptance, there is a statistically significant difference between bulgur pilafs (*p* < 0.05).

**FIGURE 7:**
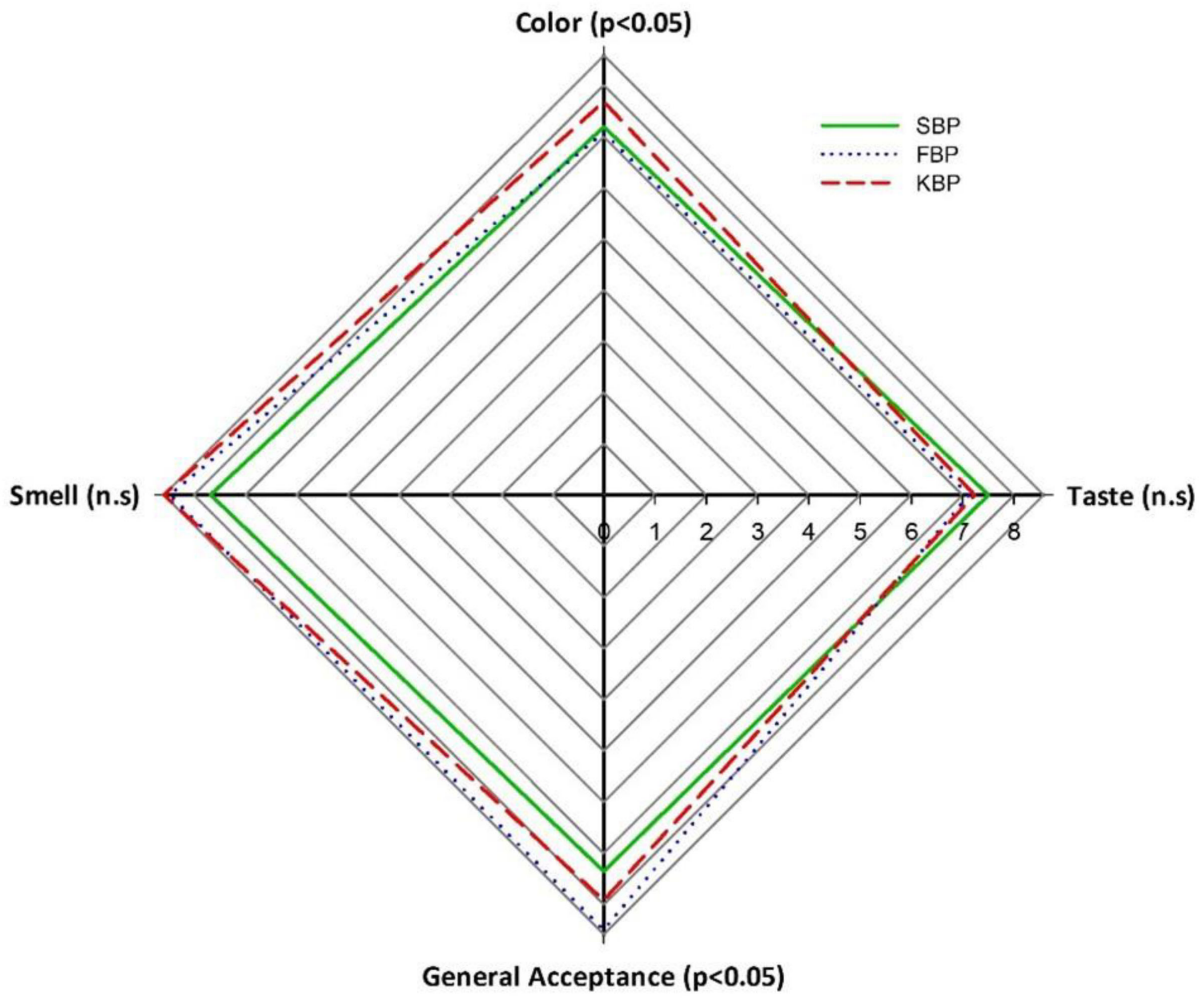
Sensory analysis graph of bulgur pilafs.

The sensory analysis graph (Figure 7) visually supports these findings and clearly shows how bulgur pilafs compare regarding different sensory properties. In particular, KBP bulgur pilaf has high values in terms of color and smell, while FBP bulgur pilaf has the highest score regarding general acceptance. These data show that SBP falls behind the other two types, especially in general acceptance, and has a lower score. These findings provide valuable information to determine the sensory properties important to consumer preferences and guide product development and marketing strategies in light of this information. In addition, statistical analyses revealed that some differences between bulgur pilafs were significant in consumer evaluations, emphasizing the sensory properties’ effect on consumer acceptance.

In a study by Yılmaz and Koca (45), sensory analysis of pilaf made from Siyez bulgur and durum bulgur found that the sensory difference between the two wheat types was due to color characteristics. They reported that this was due to the characteristic dark color of Siyez bulgur and that this situation caused Siyez bulgur pilaf to receive lower appearance and acceptability scores. The two bulgur pilafs were said to have the same taste (45).

### 3.5 Principal component analysis of bulgur pilafs

**FIGURE 8:**
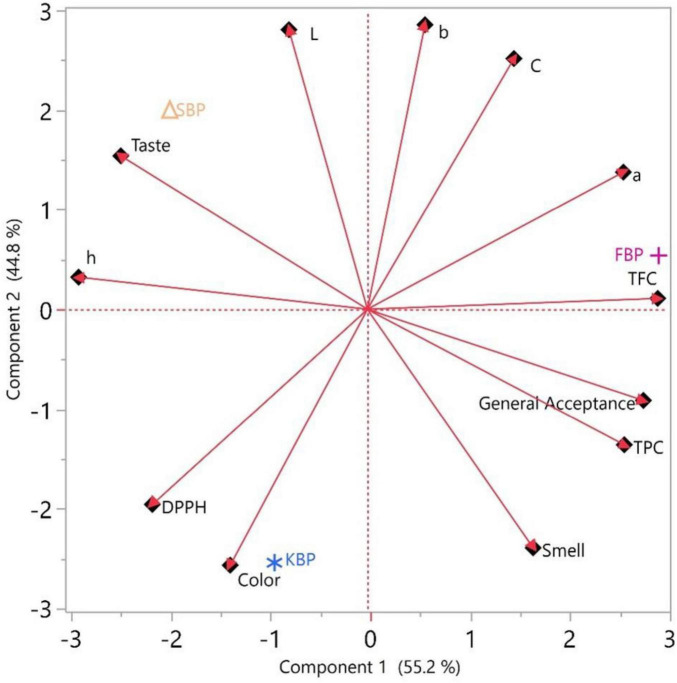
Principal component analysis (PCA) of SBP (Siyez bulgur pilafs), FBP (Firik bulgur pilafs, and KBP (Karakilçik bulgur pilafs).

**FIGURE 9:**
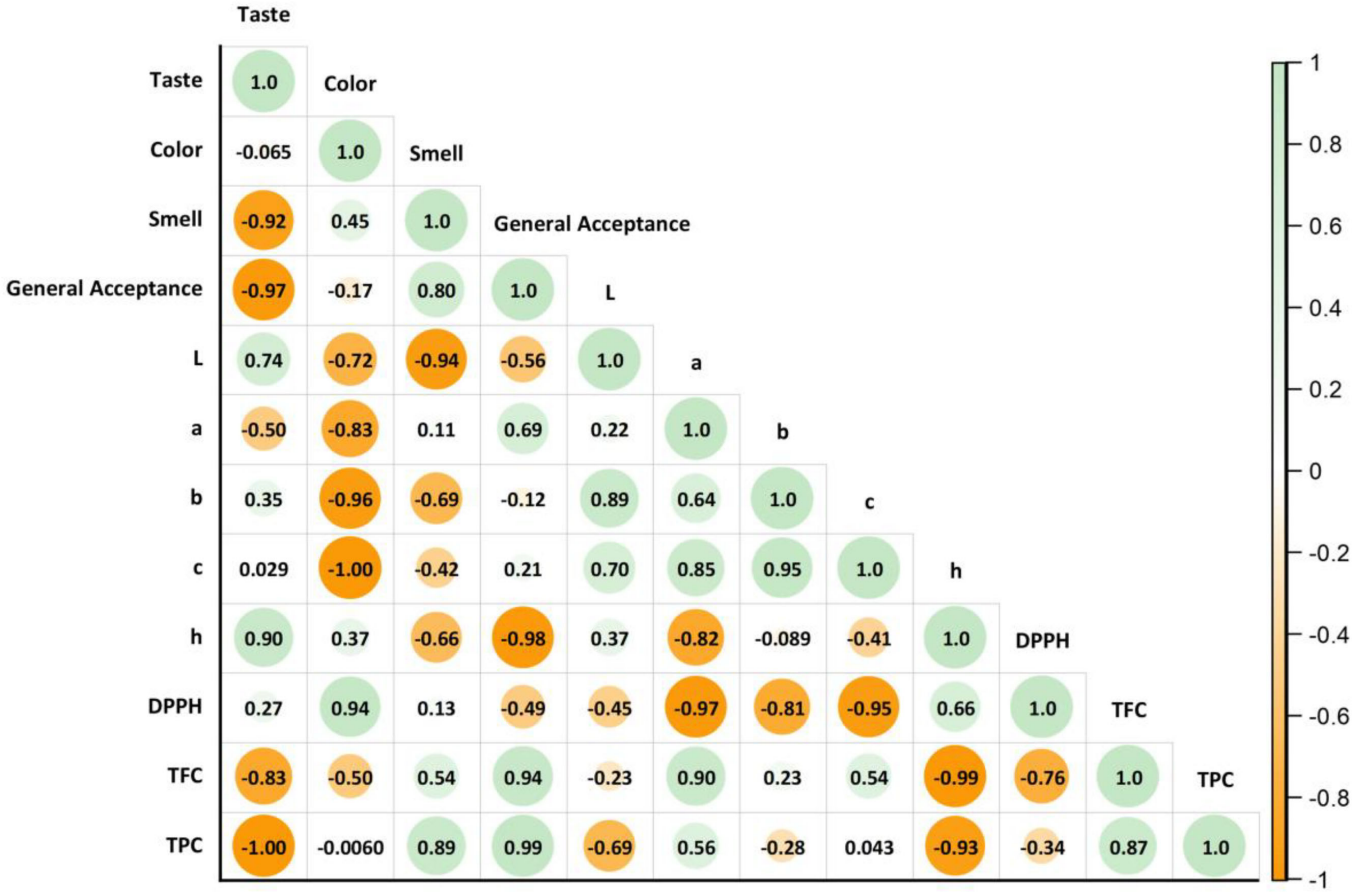
Pearson correlation relationship between sensory, color, and bioactive values of bulgur pilafs.

This study analyzed the SBP, FBP, and KBP bulgur pilafs using principal component analysis (PCA). The findings showed that the first principal component (PC1) explained 55.2% of the total variance, and the second principal component (PC2) explained 44.8% (Figure 8). Together, these two components captured 100% of the variance in the dataset and summarized all the information. SBP, FBP, and KBP bulgur pilafs were significantly clustered according to the PCA plot. This clustering indicates that these three types of bulgur pilaf are separated according to different nutritional values, physical properties, or cooking methods. According to the eigenvector analysis, the highest positive loadings on PC1 were observed for the variables “General Acceptance,” “TFC,” and “TPC.” Variables such as “Taste” and “Color” showed negative loadings. This reveals that general acceptance and total phenolic content are essential in distinguishing SBP, FBP, and KBP bulgur pilafs. On PC2, the “L,” “b,” and “C” variables showed high positive loads, while the “Smell” and “Color” variables had an adverse effect. These results show that sensory properties, such as overall acceptability, determine the differences between types and the physical properties of bulgur pilaf (such as color and smell). SBP, which is characterized by specific nutritional and physical properties, is clustered in the upper left part of the graph. FBP is clustered in the upper right and associated with general acceptance, TFC, and TPC features. KBP is clustered in the lower left and is associated with properties such as color and DPPH radical scavenging activity (DPPH). This analysis provides valuable information to identify factors essential for bulgur pilaf’s quality and consumer acceptance. It also explains how these factors can be used in product development and marketing strategies. As a result, the PCA analyses revealed how different types of bulgur pilaf differ regarding nutritional, physical, and sensory properties and which variables are responsible for these differences. This analysis provides valuable information to identify factors essential to the quality and consumer acceptance of bulgur pilafs and to understand how these factors can be used in product development and marketing strategies. These results will be helpful in future research and applications in the food industry. The distribution of samples along the PC1 and PC2 axes also reflects the quality attributes of bulgur pilafs. Samples with positive PC1 scores were associated with higher overall acceptance, TPC, and TFC values, indicating superior overall product quality. Conversely, negative PC1 scores represented lower taste and color scores, indicating decreased consumer acceptance. Similarly, positive PC2 scores were associated with higher color parameters (L, b, C), while negative scores reflected decreased sensory attributes such as odor and color. These results suggest that PCA scores visually demonstrate the combination of nutritional and sensory characteristics of bulgur pilafs and can be used as reliable indicators for defining quality and consumer acceptance. A positive correlation was found between total phenolics, total flavonoids, and antioxidant activity (Figure 9). However, a negative correlation (*r* = −0.34) was detected between TPC and DPPH activity. This finding does not constitute a contradiction, as antioxidant capacity depends not only on the total phenolic content but also on the composition of phenolic compounds and other non-phenolic bioactive compounds such as flavonoids, organic acids, and Maillard reaction products. Therefore, although TPC values are statistically similar among cultivars, significant differences in DPPH activity may occur due to differences in phenolic profiles and the contribution of non-phenolic antioxidants. This finding suggests that DPPH activity is explained not by TPC alone, but by the combined effects of multiple compounds present in the matrix.

### 3.6 PSO results and model validation

To evaluate the ability of the RSM models for each bulgur type (Siyez, Firik and Karakilçik) and to determine the optimal points, 30 independent PSO runs were performed for each of the taste, color, smell and overall acceptability objectives.

**FIGURE 10:**
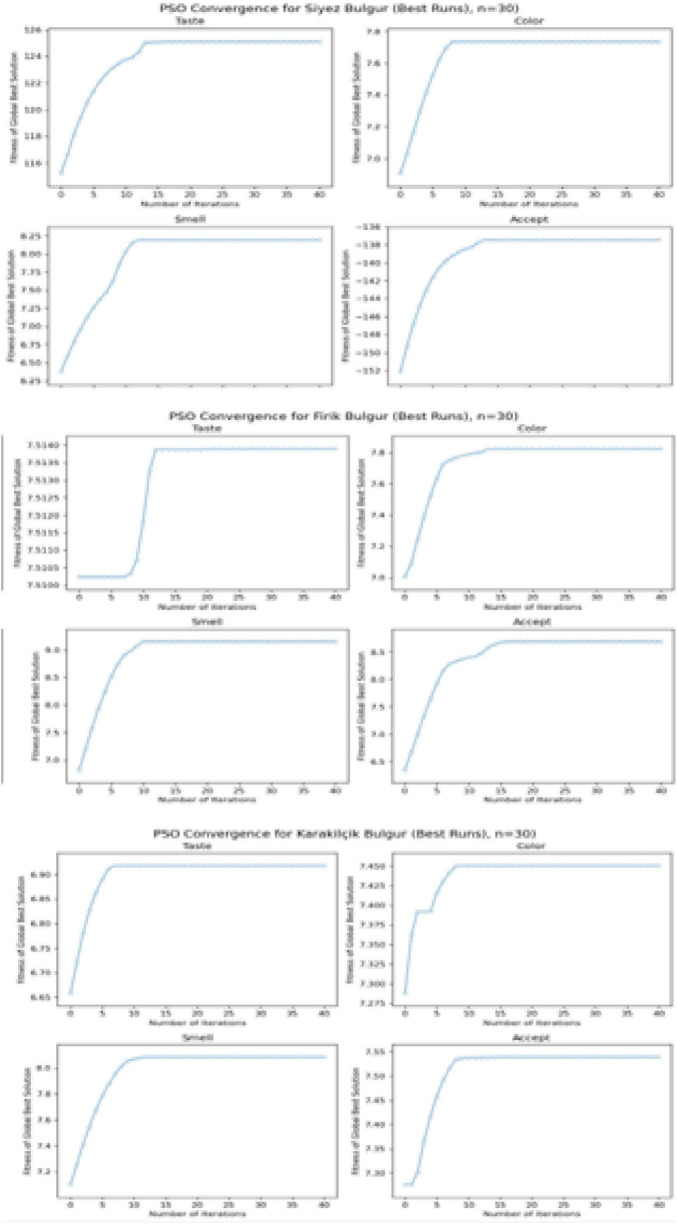
Particle Swarm Optimization (PSO) convergence curves (best runs, *n* = 30) – taste, color, smell and overall acceptability goals for Siyez, Firik and Karakilçik bulgur.

The convergence curves presented in Figure 10 show a similar trend for all taste, color, smell, and general acceptability responses for Siyez, Firik, and Karakilçik bulgur. The results show that PSO rapidly converges to the peak regions predicted by the RSM models in fewer than 40 iterations, and the curves exhibit a horizontal plateau behavior after the first 15–20 iterations. In particular, convergence occurred in less than 10 iterations for the color and smell targets, while the taste criterion showed only slight increases for all bulgur types from the 20th iteration onward.

**FIGURE 11:**
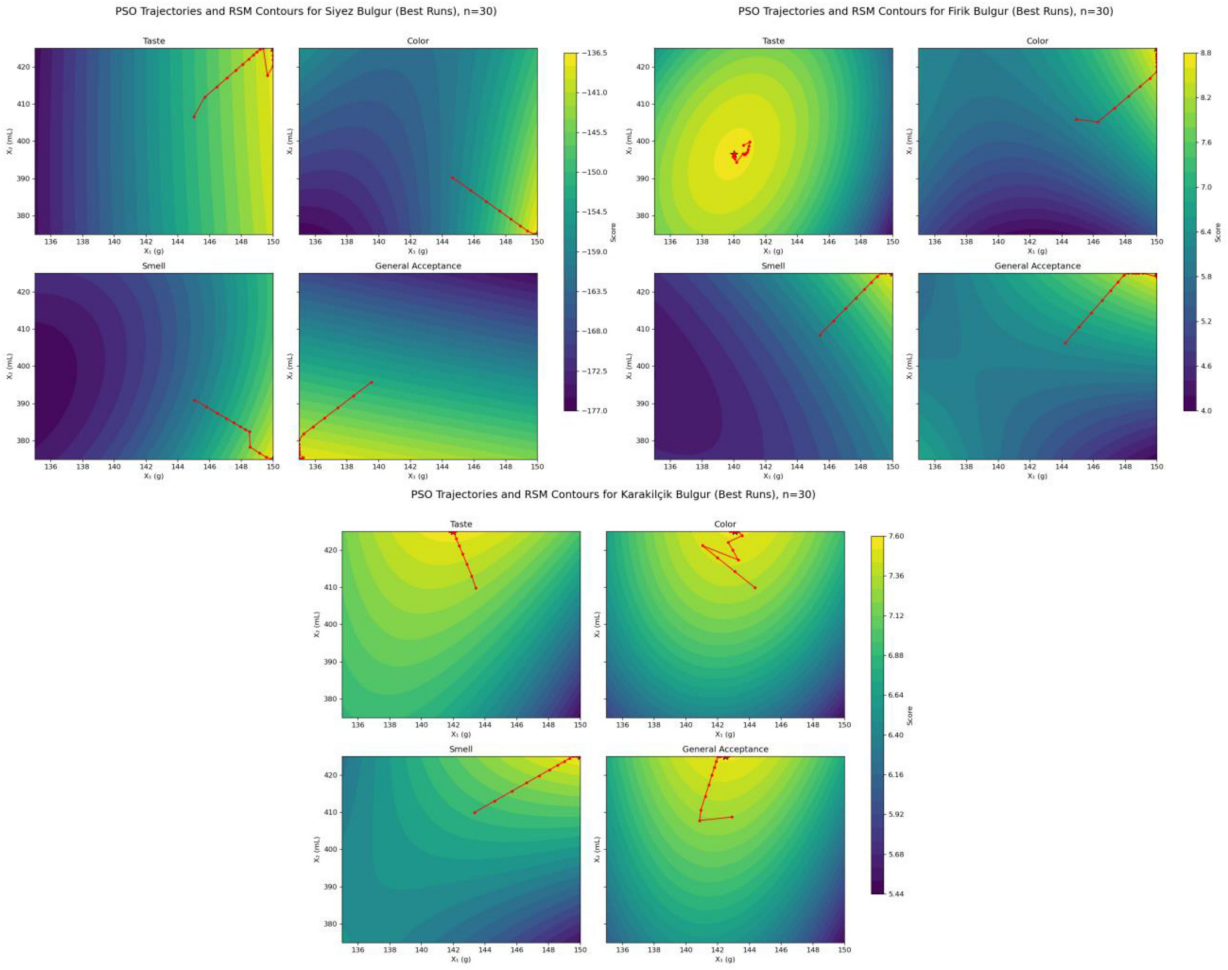
Overlay of PSO trajectories and RSM contour maps.

Figure 11 shows the PSO traces for all sensory targets (taste, color, smell, and overall acceptability) for three different bulgur types on the corresponding RSM contour maps. In each panel, the colored area represents the response values predicted by the RSM equations for the inputs X_1_ (bulgur amount, g) and X_2_ (water volume, mL); lighter shades indicate higher desirability. The red polygonal line shows the position of the global best particle selected in each iteration of the best run out of 30 independent PSO runs. In contrast, the red star indicates the final optimal point.

It can be observed that the PSO paths converge in less than 40 iterations for all bulgur types and perfectly match the bright peak areas on the RSM surfaces. This confirms the strong compatibility between empirical models and the stochastic optimization algorithm. Furthermore, the traces reveal the optimal regions specific to the sensory target. For example, while taste and color tend toward the area with high bulgur content and high water content, the overall acceptability of Siyez bulgur reaches its highest value at a lower water content. This underlines the possible need for a balance between the sensory characteristics.

Table 5 presents the optimal process parameters obtained through PSO optimization for three different bulgur types, along with a statistical summary of 30 independent runs. The optimum points for the objectives of taste, color, and smell are concentrated in the high bulgur dosage (≈ 150 g) and medium-high water level (≥375 mL) for all bulgur types. This indicates that the sensory evaluation increases with increasing bulgur content and that excessive addition of water does not bring any additional benefits. The occurrence of the general acceptability optimum at the 135 g–375 mL parameters for Siyez bulgur suggests that a lower bulgur-water combination may be beneficial in terms of consumer preference, as opposed to the taste-based optimum.

**TABLE 5:** Optimal process parameters obtained with PSO and statistical summary of 30 independent runs.

Siyez bulgur						
Optimal parameters from the best run				Multi-run statistics (*n* = 30)		
Objective	X_1_ (g)	X_2_ (mL)	Best score	Average score	Standard deviation	Best score
Taste	150.000	425.000	125.101	124.500	0.638	125.101
Color	150.000	375.000	7.735	7.539	0.116	7.735
Smell	150.000	375.000	8.195	7.551	0.392	8.195
Accept	135.000	375.000	−137.441	−139.906	2.046	−137.441
**Firik bulgur**						
Taste	140.014	396.521	7.514	7.514	0.001	7.514
Color	150.000	425.000	7.824	7.625	0.167	7.824
Smell	150.000	425.000	9.150	8.320	0.477	9.150
Accept	150.000	425.000	8.687	8.051	0.481	8.687
**Karakilçik bulgur**						
Taste	141.990	425.000	6.918	6.880	0.060	6.918
Color	143.109	425.000	7.450	7.434	0.033	7.450
Smell	150.000	425.000	8.083	7.823	0.223	8.083
Accept	142.498	425.000	7.539	7.510	0.039	7.539

X_1_ = Bulgur amount (g); X_2_ = Water (mL).

The standard deviation values reported for each objective function are extremely low (taste < 0.7; color < 0.2; smell < 0.5; overall acceptability < 0.5), suggesting that the best results obtained in 30 runs are within a small range of variation around the average value. In other words, the chosen hyperparameter set is insensitive to random initial conditions, and the algorithm produces similar optimal results in each iteration. The minimal difference between the average values and the best results underlines this finding.

As a result, the PSO-based optimization achieved both fast convergence (less than 40 iterations) and high repeatability, experimentally confirming the maximum regions predicted by the RSM model. The *X*_1_–*X*_2_ combinations used in the experimental setup, as shown in Table 5, can be recommended as reliable process conditions that can be applied on an industrial scale. The tight clustering of the final particle positions around the model peaks, as shown in Figure 11, and the slight variation between runs, as indicated in Table 5, demonstrate the robustness of the selected PSO hyperparameters and reliably confirm the predictive power of the RSM models.

## 4 Conclusion

This study demonstrated that the sensory and bioactive properties of pilafs prepared from geographically indicated Siyez, Firik, and Karakilçik bulgur varieties can be effectively optimized using RSM coupled with PSO. The results revealed distinct performance profiles: Karakilçik bulgur pilaf exhibited the highest antioxidant activity, Firik bulgur pilaf had the highest total phenolic and flavonoid contents alongside the highest general acceptability, and Siyez bulgur pilaf showed superior taste scores. These findings highlight that intrinsic compositional differences–such as protein, pigment, and phenolic compound levels–directly shape sensory perception and health-related properties.

Beyond their scientific relevance, these results have practical implications for the food industry and personalized nutrition. Firik bulgur pilaf could serve as a functional food prototype rich in bioactive compounds, Karakilçik bulgur pilaf may be positioned as a premium antioxidant-rich product, and Siyez bulgur pilaf could be developed into high-protein ready-to-eat formulations. The combined RSM–PSO approach offers a novel and reliable methodological framework to tailor product formulations based on targeted sensory and nutritional outcomes.

Overall, this study provides evidence that traditional cereals can be re-engineered through AI-assisted optimization to enhance consumer appeal and functional value. Such an approach can support innovation in the ready-to-eat food sector, promote the sustainable utilization of geographically indicated heritage grains, and contribute to healthier dietary patterns.

## Data Availability

The original contributions presented in this study are included in this article/Supplementary material, further inquiries can be directed to the corresponding authors.
