# Supplementary material for: AI-guided optimization of traditional bulgur pilafs: enhancing sensory and bioactive properties through RSM-PSO modeling
Source: Front Nutr. 2025 Oct 24;12:1658452. doi: 10.3389/fnut.2025.1658452 (PMC12591963; doi:10.3389/fnut.2025.1658452)
Supplement: Supplementary file 1 [file Table_1.docx]

**AI-Guided Optimization of Traditional Bulgur Pilafs: Enhancing Sensory and Bioactive Properties through RSM-PSO Modeling**

Sinem Türk Aslan^1^, Melikenur Türkol^2^, Seydi Yıkmış^3^, Mehmet Ali Şimşek^4^, Moneera O. Aljobair^5*^, Emad Karrar^6^, Nazlı Tokatlı^7^, Isam A. Mohamed Ahmed^8*^

**Supplementary Table 1.** Central composite test analysis of variance in a regression model (Siyez bulgur pilaf)

X_1_— Siyez bulgur; X_2_— water; df—degrees of freedom; R^2^—coefficient of determination. p<0.05, significant differences; p<0.01, very significant differences.

|  | | **Taste** | | | | **Color** | | | | | **Smell** | | | | | | **General Acceptance** | | | |
| --- | --- | --- | --- | --- | --- | --- | --- | --- | --- | --- | --- | --- | --- | --- | --- | --- | --- | --- | --- | --- |
| **Source** | **Df** | **Adj SS** | **Adj MS** | **F-Value** | **P-Value** | **Adj SS** | **Adj MS** | **F-Value** | **P-Value** | **Adj SS** | | **Adj MS** | **F-Value** | **P-Value** | **Adj SS** | | | **Adj MS** | **F-Value** | **P-Value** |
| **Model** | 5 | 166.94 | 0.33 | 43.81 | 0.000 | 119.69 | 0.24 | 61.50 | 0.000 | 633.23 | | 126.64 | 153.55 | 0.000 | 150.67 | | | 0.30 | 43.81 | 0.000 |
| **Linear** | 2 | 0.47 | 0.24 | 31.18 | 0.000 | 0.52 | 0.26 | 66.87 | 0.000 | 373.29 | | 186.64 | 226.29 | 0.000 | 0.42 | | | 0.21 | 31.18 | 0.000 |
| **X_1_** | 1 | 0.29 | 0.28 | 37.42 | 0.000 | 0.19 | 0.19 | 48.39 | 0.000 | 367.41 | | 367.41 | 445.46 | 0.000 | 0.25 | | | 0.26 | 37.42 | 0.000 |
| **X_2_** | 1 | 0.19 | 0.19 | 24.93 | 0.002 | 0.33 | 0.33 | 85.35 | 0.000 | 0.06 | | 0.05 | 414.56 | 0.032 | 0.17 | | | 0.17 | 24.93 | 0.002 |
| **Square** | 2 | 0.83 | 0.41 | 54.34 | 0.000 | 0.52 | 0.26 | 66.51 | 0.000 | 245.50 | | 122.75 | 148.83 | 0.000 | 0.74 | | | 0.37 | 54.34 | 0.000 |
| **X_1_X_1_** | 1 | 0.31 | 0.31 | 41.12 | 0.000 | 0.49 | 0.49 | 126.19 | 0.000 | 110.81 | | 110.81 | 134.35 | 0.000 | 0.28 | | | 0.28 | 41.12 | 0.000 |
| **X_2_X_2_** | 1 | 0.72 | 0.72 | 94.13 | 0.000 | 0.00 | 0.00 | 0.47 | 0.516 | 199.28 | | 199.29 | 241.62 | 0.000 | 0.65 | | | 0.65 | 94.13 | 0.000 |
| **2-Way Interaction** | 1 | 0.37 | 0.36 | 48.03 | 0.000 | 0.16 | 0.16 | 40.76 | 0.000 | 0.14 | | 0.14 | 17.51 | 0.004 | 0.33 | | | 0.33 | 48.03 | 0.000 |
| **X_1_X_2_** | 1 | 0.37 | 0.37 | 48.03 | 0.000 | 0.16 | 0.16 | 40.76 | 0.000 | 0.14 | | 0.14 | 17.51 | 0.004 | 0.33 | | | 0.33 | 48.03 | 0.000 |
| **Error** | 7 | 0.05 | 0.00 |  |  | 0.03 | 0.00 |  |  | 0.05774 | | 0.00 |  |  | 0.04 | | | 0.00 |  |  |
| **Lack-of-Fit** | 3 | 0.05 | 0.02 |  |  | 0.03 | 0.00 |  |  | 0.05 | | 0.01 |  |  | 0.04 | | | 0.01 |  |  |
| **Pure Error** | 4 | 0.00 | 0.00 |  |  | 0.00 | 0.00 |  |  | 0.00 | | 0.00 |  |  | 0.00 | | | 0.00 |  |  |
| **Total** | 12 | 172.28 |  |  |  | 122.42 |  |  |  | 639.011 | |  |  |  | 155.48 | | |  |  |  |
| **R^2^** |  | 96.90% | | | | 97.77% | | | | 99.10% | | | | | | 96.90% | | | | |
| **Adj R^2^** |  | 94.69% | | | | 96.18% | | | | 98.45% | | | | | | 94.69% | | | | |
| **Pred R^2^** |  | 69.04% | | | | 78.18% | | | | 90.80% | | | | | | 69.04% | | | | |

**Supplementary Table 2.** Central composite test analysis of variance in a regression model (Firik bulgur pilaf)

X_1_— Firik bulgur; X_2_— water; df—degrees of freedom; R^2^—coefficient of determination. p<0.05, significant differences; p<0.01, very significant differences

|  |  | **Taste** | | | | **Color** | | | | **Smell** | | | | **General Acceptance** | | | |
| --- | --- | --- | --- | --- | --- | --- | --- | --- | --- | --- | --- | --- | --- | --- | --- | --- | --- |
| **Source** | **Df** | **Adj SS** | **Adj MS** | **F-Value** | **P-Value** | **Adj SS** | **Adj MS** | **F-Value** | **P-Value** | **Adj SS** | **Adj MS** | **F-Value** | **P-Value** | **Adj SS** | **Adj MS** | **F-Value** | **P-Value** |
| **Model** | 5 | 1.91 | 0.38 | 145.20 | 0.000 | 2.35 | 0.47 | 86.07 | 0.000 | 9.84 | 1.97 | 168.26 | 0.000 | 6.06 | 1.21 | 121.05 | 0.000 |
| **Linear** | 2 | 0.05 | 0.03 | 10.25 | 0.008 | 1.14 | 0.57 | 104.14 | 0.000 | 6.62 | 3.30 | 282.96 | 0.000 | 1.47 | 0.74 | 73.60 | 0.000 |
| **X_1_** | 1 | 0.00 | 0.00 | 2.22 | 0.180 | 0.00 | 0.00 | 1.41 | 0.274 | 4.44 | 4.44 | 379.69 | 0.000 | 0.00 | 0.00 | 0.08 | 0.785 |
| **X_2_** | 1 | 0.04 | 0.05 | 18.29 | 0.004 | 1.13 | 1.13 | 206.88 | 0.000 | 2.17 | 2.17 | 186.23 | 0.000 | 1.47 | 1.47 | 147.13 | 0.000 |
| **Square** | 2 | 1.78 | 0.90 | 337.86 | 0.000 | 1.14 | 0.57 | 104.41 | 0.000 | 2.67 | 1.34 | 114.23 | 0.000 | 0.77 | 0.38 | 38.37 | 0.000 |
| **X_1_X_1_** | 1 | 1.37 | 1.37 | 520.48 | 0.000 | 0.57 | 0.57 | 104.06 | 0.000 | 1.06 | 1.06 | 91.24 | 0.000 | 0.04 | 0.04 | 4.46 | 0.073 |
| **X_2_X_2_** | 1 | 0.90 | 0.90 | 339.47 | 0.000 | 0.26 | 0.26 | 47.85 | 0.000 | 2.27 | 2.27 | 194.44 | 0.000 | 0.77 | 0.76 | 76.58 | 0.000 |
| **2-Way Interaction** | 1 | 0.08 | 0.08 | 29.80 | 0.001 | 0.07 | 0.07 | 13.22 | 0.008 | 0.54 | 0.55 | 46.94 | 0.000 | 3.82 | 3.82 | 381.31 | 0.000 |
| **X_1_X_2_** | 1 | 0.08 | 0.08 | 29.80 | 0.001 | 0.07 | 0.07 | 13.22 | 0.008 | 0.55 | 0.55 | 46.94 | 0.000 | 3.82 | 3.82 | 381.31 | 0.000 |
| **Error** | 7 | 0.01 | 0.00 |  |  | 0.03 | 0.00 |  |  | 0.08 | 0.01 |  |  | 0.07 | 0.01 |  |  |
| **Lack-of-Fit** | 3 | 0.01 | 0.00 |  |  | 0.03 | 0.01 |  |  | 0.08 | 0.03 |  |  | 0.07 | 0.02 |  |  |
| **Pure Error** | 4 | 0.00 | 0.00 |  |  | 0.00 | 0.00 |  |  | 0.00 | 0.00 |  |  | 0.00 | 0.00 |  |  |
| **Total** | 12 | 1.93 |  |  |  | 2.39 |  |  |  | 9.92 |  |  |  | 6.13 |  |  |  |
| **R^2^** |  | 99.05% | | | | 98.40% | | | | 99.17% | | | | 98.86% | | | |
| **Adj R^2^** |  | 98.36% | | | | 97.26% | | | | 98.59% | | | | 98.04% | | | |
| **Pred R^2^** |  | 91.55% | | | | 88.97% | | | | 93.18% | | | | 92.05% | | | |

**Supplementary Table 3.** Central composite test analysis of variance in a regression model (Karakilçik bulgur pilaf)

|  |  | **Taste** | | | | **Color** | | | | **Smell** | | | | | **General Acceptance** | | | |
| --- | --- | --- | --- | --- | --- | --- | --- | --- | --- | --- | --- | --- | --- | --- | --- | --- | --- | --- |
| **Source** | **Df** | **Adj SS** | **Adj MS** | **F-Value** | **P-Value** | **Adj SS** | **Adj MS** | **F-Value** | **P-Value** | **Adj SS** | **Adj MS** | **F-Value** | **P-Value** | **Adj SS** | | **Adj MS** | **F-Value** | **P-Value** |
| **Model** | 5 | 2.08 | 0.41 | 1986.57 | 0.000 | 3.69 | 0.74 | 203.86 | 0.000 | 3.21 | 0.64 | 1008.13 | 0.000 | 6.03 | | 1.21 | 492.31 | 0.000 |
| **Linear** | 2 | 1.04 | 0.52 | 2494.26 | 0.000 | 2.27 | 1.13 | 313.24 | 0.000 | 1.78 | 0.89 | 1393.40 | 0.000 | 3.05 | | 1.53 | 623.30 | 0.000 |
| **X_1_** | 1 | 0.00 | 0.00 | 45.63 | 0.000 | 0.60 | 0.60 | 166.17 | 0.000 | 0.40 | 0.40 | 632.33 | 0.000 | 0.80 | | 0.80 | 325.70 | 0.000 |
| **X_2_** | 1 | 1.03 | 1.04 | 4942.90 | 0.000 | 1.67 | 1.67 | 460.31 | 0.000 | 1.37 | 1.37 | 2154.47 | 0.000 | 2.26 | | 2.25 | 920.89 | 0.000 |
| **Square** | 2 | 0.90 | 0.45 | 2152.49 | 0.000 | 1.40 | 0.70 | 193.50 | 0.000 | 0.26 | 0.13 | 202.19 | 0.000 | 2.94 | | 1.47 | 599.53 | 0.000 |
| **X_1_X_1_** | 1 | 0.87 | 0.87 | 4163.12 | 0.000 | 1.29 | 1.29 | 357.03 | 0.000 | 0.25 | 0.25 | 394.17 | 0.000 | 2.91 | | 2.91 | 1187.50 | 0.000 |
| **X_2_X_2_** | 1 | 0.00 | 0.00 | 47.62 | 0.000 | 0.40 | 0.41 | 112.69 | 0.000 | 0.05 | 0.05 | 75.73 | 0.000 | 0.10 | | 0.10 | 42.56 | 0.000 |
| **2-Way Interaction** | 1 | 0.13 | 0.13 | 639.32 | 0.000 | 0.021 | 0.021 | 5.79 | 0.047 | 1.18 | 1.18 | 1849.49 | 0.000 | 0.03 | | 0.03 | 15.89 | 0.005 |
| **X_1_X_2_** | 1 | 0.13 | 0.13 | 639.32 | 0.000 | 0.021 | 0.021 | 5.79 | 0.047 | 1.18 | 1.18 | 1849.49 | 0.000 | 0.03 | | 0.03 | 15.89 | 0.005 |
| **Error** | 7 | 0.00 | 0.00 |  |  | 0.02 | 0.00 |  |  | 0.00 | 0.00 |  |  | 0.01 | | 0.00 |  |  |
| **Lack-of-Fit** | 3 | 0.00 | 0.00 |  |  | 0.02 | 0.00 |  |  | 0.00 | 0.00 |  |  | 0.01 | | 0.00 |  |  |
| **Pure Error** | 4 | 0.00 | 0.00 |  |  | 0.00 | 0.00 |  |  | 0.00 | 0.00 |  |  | 0.00 | | 0.00 |  |  |
| **Total** | 12 | 2.08 |  |  |  | 3.72 |  |  |  | 3.21 |  |  |  | 6.05 | |  |  |  |
| **R^2^** |  | 99.93% | | | | 99.32% | | | | 99.86% | | | | | 99.72% | | | |
| **Adj R^2^** |  | 99.88% | | | | 98.83% | | | | 99.76% | | | | | 99.51% | | | |
| **Pred R^2^** |  | 99.29% | | | | 95.04% | | | | 98.74% | | | | | 98.06% | | | |

X_1_— Karakilçik bulgur; X_2_— water; df—degrees of freedom; R^2^—coefficient of determination. p<0.05, significant differences; p<0.01, very significant differences
